# HIF-1α/BNIP3-Mediated Autophagy Contributes to the Luteinization of Granulosa Cells During the Formation of Corpus Luteum

**DOI:** 10.3389/fcell.2020.619924

**Published:** 2021-01-18

**Authors:** Zonghao Tang, Zhenghong Zhang, Qingqiang Lin, Renfeng Xu, Jiajie Chen, Yuhua Wang, Yan Zhang, Yedong Tang, Congjian Shi, Yiping Liu, Hongqin Yang, Zhengchao Wang

**Affiliations:** ^1^Provincial Key Laboratory for Developmental Biology and Neurosciences, Provincial University Key Laboratory of Sport and Health Science, Key Laboratory of Optoelectronic Science and Technology for Medicine of Ministry of Education, College of Life Sciences, Fujian Normal University, Fuzhou, China; ^2^Key Laboratory of Medical Electrophysiology of Ministry of Education and Sichuan Province, Drug Discovery Research Center, Southwest Medical University, Luzhou, China; ^3^Department of Pharmacological and Pharmaceutical Sciences, College of Pharmacy, University of Houston, Houston, TX, United States; ^4^Provincial Key Laboratory of Reproductive Health Research, School of Medicine, Xiamen University, Xiamen, China

**Keywords:** corpus luteum, cell apoptosis, luteinization, granulosa cell, autophagy, HIF-1α/BNIP3

## Abstract

During the luteinization after ovulation in mammalian ovary, the containing cells undergo an energy consuming function re-determination process to differentiate into luteal cells under avascular environment. Previous evidences have delineated the contribution of autophagy to the cell differentiation and the catabolic homeostasis in various types of mammalian cells, whereas few interest had been focused on the involvement of autophagy in the luteinization of granulosa cells during the formation of early corpus luteum. Herein, the present study investigated that expression and contribution of autophagy during granulosa cell luteinization and early luteal development through *in vivo* and *in vitro* experiments. The results clearly demonstrated that HIF-1α/BNIP3-mediated autophagy plays a vital role in the luteinization of granulosa cells during the early luteal formation *in vivo* and *in vitro*. In the neonatal corpus luteum, HIF-1α up-regulated BNIP3 expressions, which contributed to the autophagic initiation by disrupting beclin1 from Bcl-2/beclin1 complex and protected cells from apoptosis by curbing the skew of mitochondria balance under avascular niche. Notably, Inhibition of HIF-1α activity by echinomycin enhanced the levels of cytoplasmic cytochrome c and cell apoptosis in the nascent corpus luteum. These findings revealed that HIF-1α/BNIP3-mediated autophagy enabled the process of granulosa cell luteinization and protected the granulosa-lutein cells from further apoptosis under hypoxia niche. To our knowledge, the present study firstly clarified that HIF-1α/BNIP3-mediated autophagy contributes to the luteinization of granulosa cells during the formation of pregnant corpus luteum, which will help us further understanding the luteal biology and provide us new clues for the treatment of luteal insufficiency.

## Introduction

Corpus luteum is an ephemeral endocrine gland responsible for the secretion of sexual hormones and the maintenance of mammalian pregnancy (Galvão et al., 2013). It is widely acknowledged that corpus luteum is evolved from the remainder of ovarian follicle ovulated, the evolution of this process is termed as luteinization. The synchronized sub-cellular reorganization of granulosa cells is decisive to the determination of luteal cells and the formation of corpus luteum. Notably, there exist significant environmental differences between granulosa cell and other types of cells during the differentiation. As the oxygen concentration in follicular fluid is decreased with the development of follicles, evolving a hypoxia niche is prepared for thereafter physiological activities (Fischer et al., 1992). Although the contracted corpus luteum undergoes a rapid and remarkable amount of tissue remodeling and angiogenesis, the formation of luxurious blood vessel network consumes a few days after ovulation (Davis et al., 2003; Stocco et al., 2007). Thus, both the differentiation of granulosa cell and the formation of corpus luteum are progressed under low oxygen conditions (Huey et al., 1999). Canonically, the hypoxia condition negatively affects cell metabolism and may also hamper cellular functions, whereas maintaining a hypoxia environment is meaningful to the functions of granulosa cells, indicating different physiological status exist between follicular cells and other types of cells (van den Driesche et al., 2008). Recent studies revealed that the establishment of hypoxia condition is merit to granulosa cell differentiation by a HIF-1α-dependent pathway in mouse ovary (Chen et al., 2017). However, by which mechanism granulosa cells overcome hypoxia condition to initiate the program of cell differentiation still remains elusive.

Autophagy is an evolutionarily conserved self-eating program, which recycles long-lived proteins and organelles under adverse conditions to alleviate metabolic stresses and maintain the homeostasis of cell metabolism (Galluzzi et al., 2014). In mammalian ovary, although luteal cells are differentiated from pre-exist granulosa cells (Galvão et al., 2013), there exist significant discrepancies between luteal cells and their precursor granulosa cells, including the vary of hormonal receptors, enzymes and morphologies (Niswender et al., 2000). The complement of luteinization requires the switch of cell metabolic traditions, the change of cellular cytoskeleton, the shift of cell-dependent hormones and the modification of cellular functions during the process of granulosa cell differentiation (Murphy, 2000). Thus, it is necessary for cells to induce autophagy to remolding itself morphologies and reprogramming metabolic activities during the differentiation (Ozturk et al., 2017). Expectedly, previous investigations have revealed the involvement of autophagy in cell differentiation among various types of cells, including myeloid cells (Wang et al., 2011), muscle cells (Iovino et al., 2012), neuron cells (Agostini et al., 2016), adipogenic stem cells (Drehmer et al., 2016), cardiac stem cells (Zhang et al., 2012), and retinal ganglion cells (Esteban-Martínez and Boya, 2018). Noteworthy, autophagic program is also necessary for spermatid differentiation and acrosome biogenesis during the spermatogenesis of male testes (Wang et al., 2014; Shang et al., 2016). The ubiquitous involvement of autophagy during cell reprogramming hints us whether the induction of autophagy is an inherent mechanism available for mammalian cell differentiation.

Given the differentiation of granulosa cells and the formation of corpus luteum are undertaken under hypoxia environment, we thereafter hypothesized whether hypoxia-inducible factor (HIF)-1α is a crucial factor in autophagic regulation during this physiological process. Therefore, the present study was designed to investigate the induction of autophagy during the luteinization of granulosa cells and the formation of early corpus luteum. In additional, the involvement of HIF-1α pathway in autophagic regulation was also examined *in vivo* and *in vitro* by echinomycin, a HIF-1α specific inhibitor. The present results will clarify the molecular mechanism regulating the luteinization of granulosa cells during the formation of pregnant corpus luteum, which will help us further understanding the luteal biology and provide us new clues for the treatment of luteal insufficiency.

## Methods

### Animals

The female Sprague–Dawley rats (about 250 g body weight) were purchased from Wushi Experimental Animal Supply Co., Ltd. (Fuzhou, China), and these rats were allowed to accommodate for 1 week prior to mating with males in Laboratory Animal Center of Fujian Normal University. Two female rats were housed with one male and the presence of a vaginal plug were examined every morning. The mating of rats was scheduled according to the demand of experiments. Day 1 of pregnancy was defined as the day when a vaginal plug was recovered. To examine the possible role of HIF-1α signaling during the formation of corpus luteum from the pregnant rats, echinomycin (Sigma-Aldrich, 75 μg/kg), a potent inhibitor of HIF-1α (Foster et al., 1985), was intraperitoneally injected before mating or execution. The dosage of echinomycin treatment is gentler than those used in human clinical trials, whereas can effectively inhibit the binding activity of HIF-1α. In addition, to evaluate the effect of the inhibition of autophagy on apoptosis during the formation of corpus luteum, we also treated the rats with chloroquine (CQ, 30 mg/kg body weight) before mating. All samples were executed at designed time points and the ovarian samples were harvested for following experiments. Briefly, ovaries were fixed in 4% paraformaldehyde or immediately suffered for co-immunoprecipitation, mitochondrial extraction and snap-frozen. Animal experimental protocols of present study were reviewed and approved by the Ethics Committee on Animal Experimentation of Fujian Normal University.

### Immunohistochemistry and Immunofluorescence

The whole paraffin-embedded ovarian sections were de-paraffinized and re-hydrated. Thereafter, the sections were subjected to antigen microwave antigen retrieval by 0.01 M citric acid buffer for 10 min. Endogenous peroxide was regularly reduced by incubating the sections in 3% H_2_O_2_ for 20 min. For non-specific binding inhibition, the sections were blocked with 5% BSA in PBS for 30 min. After washing, the sections were incubated overnight at 4°C with anti-LC-3I/II antibody (diluted 1:100, Abcam, Cambridge, MA, USA), anti-Beclin1 antibody (1:100 dilution, Protein Tech Group, Wuhan, China) and anti-cleaved caspase-3 antibody (Cell Signaling Technology, Boston, MA, USA). After washing with PBS three times, these slides were incubated with the secondary antibodies at room temperature for 30 min. Diaminobenzidine tetrahydrochloride chromogen staining was applied for visualization. All sections were thereafter counterstained with hematoxylin, dehydrated, and mounted lastly.

The formation of autophagosomes in cells were detected by immunofluorescence. After treatment, cells were washed with ice-cold PBS followed by methyl alcohol fixation for 10 min. Then, cells were treated with 0.5% TritonX-100 for 5 min. After LC3 antibody incubation, cells were incubated with secondary antibody (Alexa Flour 594). Finally, slides were mounted and observed under a confocal laser scanning microscope (Carl Zeiss, Göttingen, Germany).

### Cell Culture and Treatment

The ovaries from rats on the day of estrus were excised and placed in DMEM/F12 medium (Hyclone) supplemented with 10% fetal bovine serum (Hyclone), 10 mg/ml streptomycin sulfate and 75 mg/ml penicillin G (Hyclone). Granulosa cells were harvested from follicles by using a 25-gauge needle. After follicle puncture, granulosa cells were collected, washed and suspended in the appropriate solution for western blot analysis or further culture maintained in a humidified 5% CO_2_ environment at 37°C (Tam et al., 2010). Further treatments were launched after cell adherence and hCG (10 IU /ml, Ningbo Second Hormone Factory, Ningbo, China) were employed to induce granulosa cell differentiation. To evaluate the role of HIF-1α pathway during cell differentiation, CoCl_2_ (100 μM Sigma-Aldrich) was added to inhibit HIF-1α degradation in these cells. The role of autophagy in cell differentiation was verified by the treatment of 3-MA (10 mM, Sigma).

### Transfected Cells With siRNA

Granulosa cells were plated the day before transfection to achieve ~50% confluency, and then transiently transfected using Lipofectamine 3000 (Invitrogen) according to the protocol provided by the manufacturer. Non-targeting siRNA and siRNA targeted against beclin1 and BNIP3 were purchased from Genepharma (Shanghai). For each transfection, 200 nmols of siRNA was added per 10 cm plate at a concentration of 40 nM. 48 h after transfection, cells were seeded into 6-well plates for drug treatments. The sequences of siRNAs were listed as follows (Prabhakaran et al., 2007): beclin1 siRNA, sense, 5′-CUC AGG AGA GGA GCC AUU UTT-3′, anti-sense, 5′-AAA UGG CUC CUC UCC UGA GTT-3′, BNIP3 siRNA, sense, 5′-GCU GCC CUG CUA CCU CUC ATT-3′ and anti-sense 5′-UGA GAG GUA GCA GGG CAGC TT-3′, and negative control siRNA, sense, 5′-UUC UCC GAA CGU GUC ACG UTT-3′, anti-sense, 5′-ACG UGA CAC GUU CGG AGA ATT-3′.

### Cell Apoptosis Assay

For cell apoptosis evaluation, the corpus luteum was disassociated from rat ovaries on the specific day after pregnancy under dissecting microscope according to the method described by Care et al. (2013) with minor modifications. Briefly, the corpus luteum was trimmed of fat and minced into small pieces before enzymatic digestion into single cells. The minced tissues were incubated for 1 h at 37°C in DMEM F12 containing 0.1% collagenase A (Gibco), dispase (Gibco), and 25 μg/ml DNase 1 (Sigma-Aldrich). The cells were passed through a 70-μm nylon strainer (Becton Dickinson, Franklin Lakes, NJ, USA) to remove debris, and the filtrate was centrifuged at 300 g and 4°C for 5 min to pellet the suspended cells. After that, the cells were washed in FACS buffer (PBS containing 0.1% BSA). Thereafter, cells were re-suspended and stained by Annexin V-FITC Apoptosis Detection Kit (Beyotime Institute of Biotechnology, Haimen, China) according to the protocol provided by the manufacturer. The apoptotic cells were thereafter measured by using a BD FAC Symphony A5 system (Becton Dickinson, Franklin, NJ, USA).

### JC-1 Staining

In order to assess mitochondrial status, the present study tracked relative mitochondrial transmembrane potential through detecting JC-1 fluorescence. The lipophilic cation JC-1 could reversibly changes its fluorescence from green (monomeric status) to red (multimeric status) according to the variation of mitochondrial potential. JC-1 kit (Beyotime Institute of Biotechnology) was used to evaluate the mitochondrial status of luteal cells separated from the corpus luteum according to the methods provided by the manufacturer. Briefly, the separated luteal cells were digested into single cell suspension, collected and incubated with 10 μg/ml of 5, 5′, 6, 6′-tetrachloro-1, 1′, 3, 3′-tetraethylimidacarbocyanine iodide (JC-1) at 37°C, 5% CO_2_ for 30 min. After washing, cells were analyzed by using a BD FACSymphony A5 system (Becton Dickinson, Franklin, NJ, USA).

### Western Blot Analysis

The corpus luteum was separated from the ovaries under a dissecting microscope with great care. In order to examine the expression of autophagy related proteins, the isolated corpus luteum was homogenized and total lysates by ice-cold RIPA buffer with supplemented protease inhibitors (Protease inhibitor cocktail, Beyotime Institute of Biotechnology, Haimen, China). The prepared yield lysates (30 mg protein/lane) were loaded by SDS-PAGE and then transferred to polyvinylidene difluoride (PVDF) membranes (Pall Life Sciences, Port Washington, NY, USA). The non-specific binding was blocked by 5% skim milk, and the membranes were thereafter incubated overnight in the presence of primary antibodies (Supplementary Table 1). After washing with TBST, the membranes were incubated in horseradish peroxidase-conjugated goat anti-rabbit or anti-mouse IgG (1:1000 dilution, Beyotime Institute of Biotechnology, Haimen, China) for 1 h at room temperature. The bands were visualized using enhanced chemiluminscence star (ECL, Beyotime Institute of Biotechnology, Haimen, China). The blots were quantified using ImageJ 1.49 software (National Institutes of Health, Bethesda, MD, USA).

### RNA Extraction and Real-Time PCR Analysis

Total mRNA was extracted from the isolated corpus luteum by TRIzol solution (Invitrogen Life Technologies, Carlsbad, CA, USA) according to the instructions provided by manufacture. The extracted mRNA samples were immediately applied for reverse-transcription by a cDNA Synthesis kit (Promega, Biotech Co., Ltd). The reverse-transcribed products were amplified using a FasQuant RT Kit (Tiangen Biotech Co., Ltd, Beijing, China), with Go Taq qPCR Master Mix (Promega corporation, Lot 0000209928). Primers specific for BNIP3 (forward primer 5′-CTC TGC TGA GTG AAG TTC TAC G-3′, reverse primer 5′-AAC ACA AGT GCT GGA TAC TGA TT-3′), NIX (forward primer 5′-GCA GTG CCA TTG AAC TGT GG-3′, reverse primer 5′-GGA ACC GCA AAT CGA CAT CG-3′) Atg5 (forward primer 5′-AGA AGA AGA GCC AGG TGA TGA-3′, reverse primer 5′-AAT GCT GAT GTG AAG GAA GTT GT-3′), LC-3I/II (forward primer 5′-CCT CTG CTT CCT GCT ACC T-3′, reverse primer 5′-GTG GCT GTG TTG GCT TCC-3′), and beclin1 (forward primer 5′-ATG CTGT CCT TTC CCT CTT CC-3′, reverse primer 5′-ACC TTT ACC TCT TGT CCC TTC C-3′) were designed and commercially synthesized. A total 20 μl PCR reaction mix was used, including 10.0 μl 2X Go Taq qPCR Master Mix (Promega corporation), 0.2 ul CXR reference Dye, 2.0 μl cDNA template, 7.0 μl RNase-free water and 0.8 ul primers (containing 0.4 ul forward and 0.4 ul reverse). The reaction procedure of RT-qPCR system (Applied Biosystems Life Technologies) was set as follow: 50°C for 2 min, 95°C for 10 min, followed by 40 cycles at 95°C for 15 s, and 60°C for 1 min. The relative gene expression levels were calculated in accordance with the ΔΔCt method, and relative mRNA levels were expressed as 2^−ΔΔCt^ values (Zhang et al., 2015).

### Co-immunoprecipitation

To explore protein-protein interactions in the corpus luteum, fresh luteal samples were regularly homogenized using 0.5 ml of immunoprecipitation lysis buffer with the presence of proteinase inhibitors (Protease inhibitor cocktail, Beyotime Institute of Biotechnology, Haimen, China). The Lysates were clarified by centrifugation at 4,000 g for 10 min at 4°C, and then the supernatants were collected. The concentration of samples was measured using BCA kit (Beyotime Institute of Biotechnology, Haimen, China) and then 20 ug protein of each sample was removed as input and the remainder was precleared by Protein A/G PLUS-agarose (Santa Cruz Biotechnology; SC 2003) for two times, species matched IgG antibodies were employed as controls (Beyotime Institute of Biotechnology, Haimen, China). Samples were then incubated at 4°C overnight with the presence of specific antibodies. Thereafter, Protein A/G PLUS-agarose (Santa Cruz Biotechnology; SC-2003) was used as anchor for complex binding. After centrifugation (4,000 g for 5 min at 4°C), the agarose beads were collected and washed by rotating 5 min at 4°C with PBS for four times. Bound proteins were collected in SDS sample buffer and subjected to SDS-PAGE and Western blotting.

### Transmission Electron Microscopy

Luteal tissues were collected from the ovaries of pregnant rats with or without echinomycin treatment. Specimens for transmission electron microscopy were prepared and fixed with 2.5% (vol/vol) glutaraldehyde (Solarbio, P1126) in PBS (4°C, pH 7.4, 0.1 M) for 24 h. Samples were then post-fixed with 1% OsO_4_ (Ted Pella) for 1.5 h. Graded alcohol series were thereafter used for dehydration, samples were embedded in Araldite (SPI, 90529-77-4), sectioned to ~60 nm, and mounted on Formvar-coated grids (Ted Pella, 01700-F). The ultrathin sections were contrasted with 3% aqueous uranyl acetate and lead citrate staining, examined and photographed under a transmission electron microscope (JEM-2100, Japan).

### Statistical Analysis

All experimental values were presented as mean ± SE. The significant differences in the mean values within or between treatment groups were evaluated by one-way analysis of variance, followed by Tukey's multiple range test. Statistical analysis was conducted using SPSS version 20. Statistical significant difference was inferred at *P* < 0.05.

## Results

### HIF-1α Is Involved in the Luteinization of Granulosa Cells

**Figure 1:**
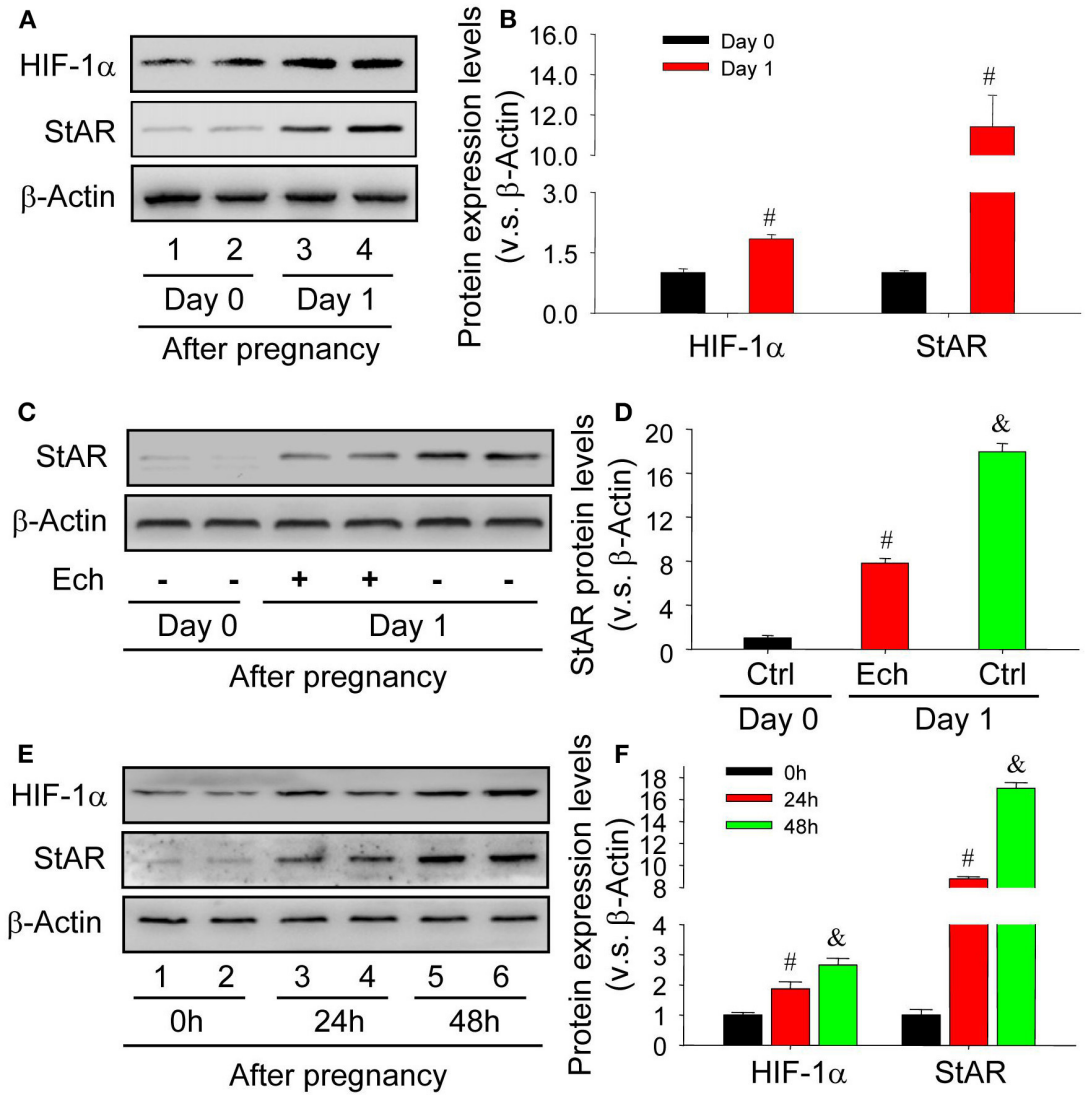
HIF-1α expression is concomitantly induced during the newly formation of corpus luteum. **(A)** Western analysis of HIF-1α and StAR expressions on Day 0 and 1 after pregnancy. **(B)** Summarized intensities of HIF-1α and StAR blotting normalized to the control (Day 0). **(C)** The expression changes of StAR in the presence or absence of echinomycin treatment on Day 1 after pregnancy. **(D)** Summarized intensities of StAR blotting normalized to the control (Day 0). **(E)** The expression changes of HIF-1α and StAR in luteinizing granulosa cells wtih hCG and CoCl2 treatment. **(F)** Summarized intensities of StAR blots normalized to the control (0 h). Each value represents the mean ± SE. One-way analysis of variance (ANOVA) was used to analyze the data, followed by a Tukey's multiple range test. *n* = 6. ^#^*P* < 0.05, vs. the group in black; ^&^*P* < 0.05, vs. the group in red.

The activation of HIF-1α pathway under hypoxia condition is important to the maintenance of cell homeostasis. During the luteinization of granulosa cells, the engaged components were suffered from insufficient oxygen supply (Kim et al., 2009). The results of *in vivo* experiments demonstrated that the level of HIF-1α was concomitantly increased with StAR, the marker of granulosa cell luteinization (Figures 1A,B). In additional, inhibition of HIF-1α activity by echinomycin significantly hampered the luteinization of granulosa cells *in vivo* (Figures 1C,D). To further confirm the role of HIF-1α during the luteinization, *in vitro* experiments were launched and found that the increase of HIF-1α expression promoted the enhancement of StAR protein level in a time-dependent manner (Figures 1E,F). These findings indicated that HIF-1α was involved in the luteinization of granulosa cells *in vivo* and *in vitro*.

### Autophagy Is Required for the Luteinization of Granulosa Cell

**Figure 2:**
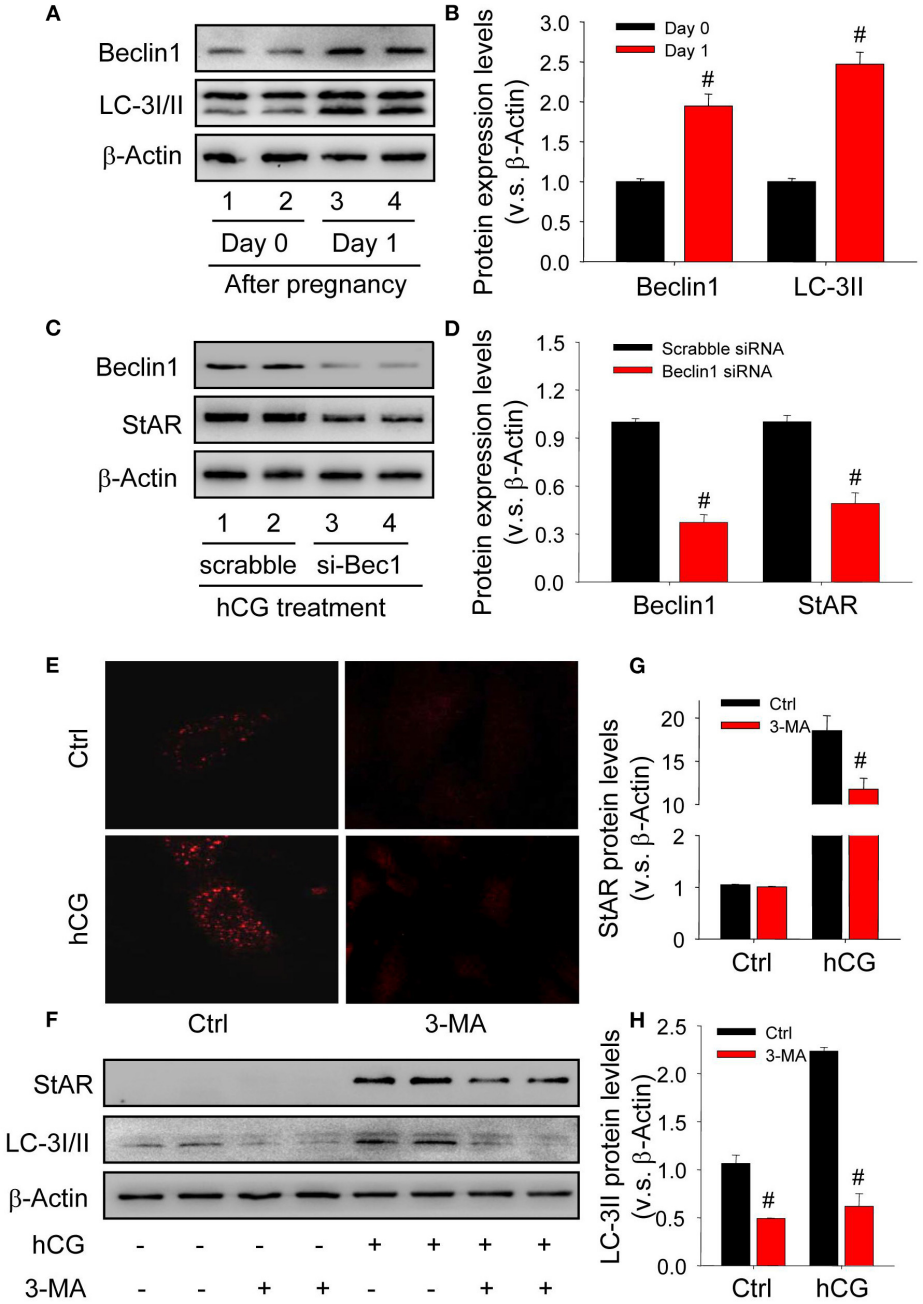
Autophagy is induced during the luteinization of granulosa cells *in vivo* and *in vitro*. **(A)** The expressions of autophagy marker proteins LC-3I/II and beclin1 on Day 0 or 1 after pregnancy were detected by Western blot. **(B)** Summarized intensities of StAR blotting normalized to the control (Day 0). **(C)** Granulosa cells were transfected with siRNA targeting beclin1 for 36 h, and then treated with hCG for 48 h. The expressions of beclin1 and StAR were analyzed by Western blot. **(D)** Summarized intensities of beclin1 and StAR blotting normalized to the control (scrabble siRNA). **(E)** Immunofluorescence of LC-3I/II in granulosa cells was pretreated with/without hCG and then treated with/without 3-MA for 24 h. **(F)** The expression changes of LC-3I/II and beclin1 detected by Western blot in granulosa cells pretreated with/without hCG (15 IU/ml) for 48 h and then treated with 3-MA (10 mM) for 24 h before analysis. **(G)** Summarized intensities of StAR blotting normalized to the control. **(H)** Summarized intensities of LC-3II blotting normalized to the control. Each value represents the mean ± SE. One-way analysis of variance (ANOVA) was used to analyze the data, followed by a Tukey's multiple range test. *n* = 6. Ctrl, vehicle; scrabble, scrabble siRNA; si-Bec1, siRNA targeting beclin1; hCG, human chorionic gonadotropin; 3-MA, 3-methyladenine, ^#^*P* < *0.05*, vs. the group in black.

Given autophagy is crucial for the transformation of cellular morphology (Mizushima and Levine, 2010), the present study thus examined whether autophagy was induced and required during the luteinization of granulosa cells. The results of *in vivo* study showed the expressions of autophagy marker proteins were significantly induced during the luteinization (Figures 2A,B), and the results of *in vitro* experiments also revealed that knock-down beclin1 by beclin1-specific small RNA interference compromised the levels of StAR, indicating an essential role of autophagy during the luteinization of granulosa cells (Figures 2C,D). In addition, hCG treatment obviously increased the content of autophagosomes in cells, while chloroquine (CQ) treatment further increased the accumulatio of autophagosomes (Supplementary Figure 1). Consistently, inhibition of autophagy by the specific inhibitor 3-methyladenine (3-MA) also contributed to the dwindled StAR levels in hCG-treated granulosa-lutein cells compared with the control (Figures 2E–H). These findings revealed that the induction of autophagy is required for the luteinization of granulosa cells *in vivo* and *in vitro*.

### HIF-1α Regulates the Activation of Autophagy in a BNIP3-Dependent Manner During the Luteinization of Granulosa Cells

**Figure 3:**
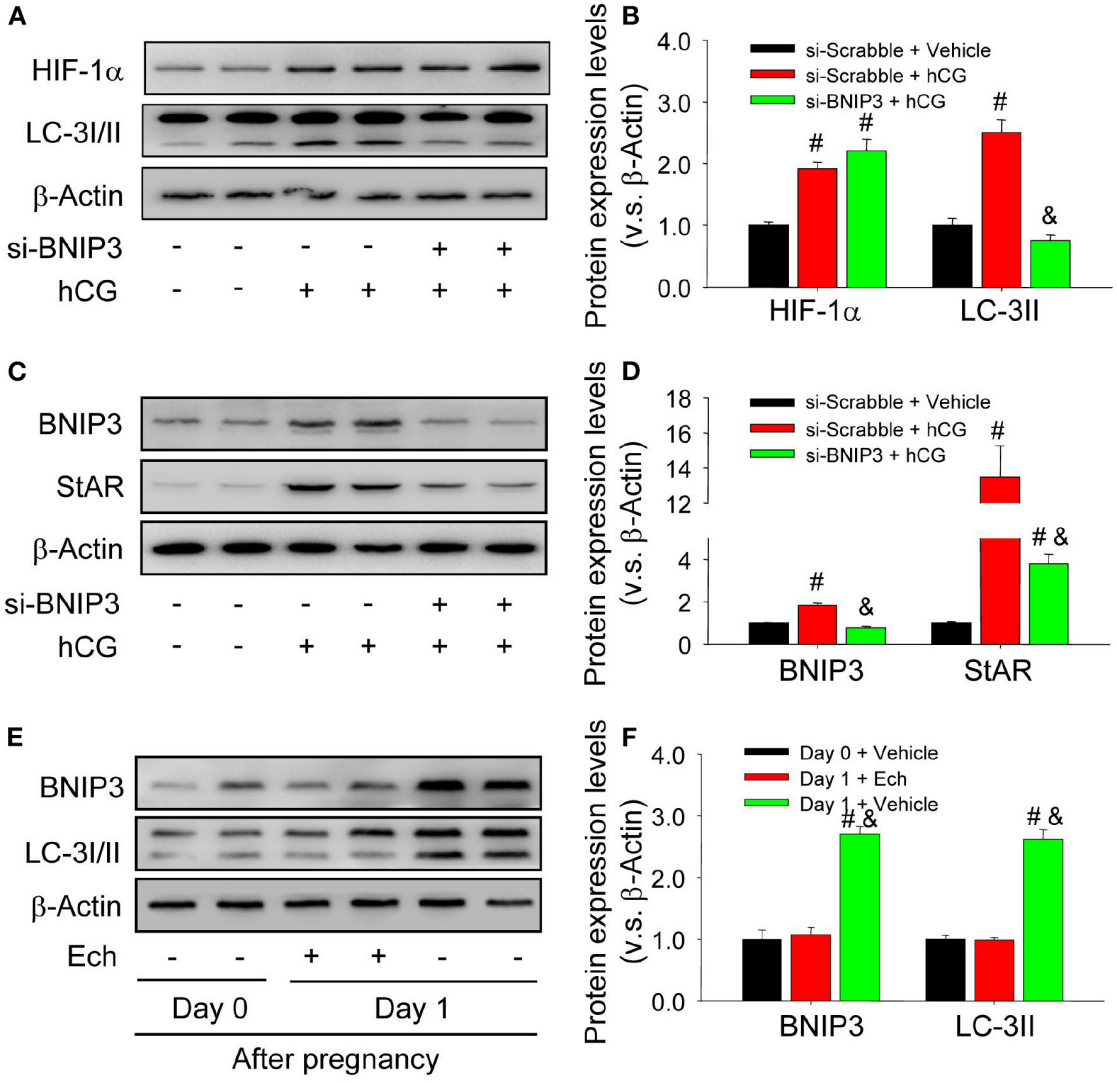
Autophagy is regulated by HIF-1α/BNIP3 pathway. **(A)** The expression changes of HIF-1α and LC-3I/II in granulosa cells transfected with si- BNIP3 for 36 h after hCG treatment. **(B)** Summarized intensities of HIF-1α and LC-3I/II blotting normalized to the control (si-scrabble and vehicle). **(C)** The expression changes of BNIP3 and StAR in granulosa cells transfected with si- BNIP3 for 36 h after hCG treatment. **(D)** Summarized intensities of BNIP3 and StAR blotting normalized to the control (si-scrabble and vehicle). **(E)** The expression changes of BNIP3 and LC-3I/II during the formation of corpus luteum after echinomycin treatment by Western blot. **(F)** Summarized intensities of BNIP3 and LC-3I/II bloting normalized to the control (Day 0). Each value represents the mean ± SE. One-way analysis of variance (ANOVA) was used to analyze the data, followed by a Tukey's multiple range test. *n* = 6. si-scrabble, scrabble siRNA; si- BNIP3, siRNA targeting BNIP3; hCG, human chorionic gonadotropin; Ech, echinomycin, ^#^*P* < 0.05, vs. the group in black; ^&^*P* < 0.05, vs. the group in red.

To clarify the mechanism underlying the above-demonstrated activation of autophagy during the luteinization, the present study thus examined the expression of BNIP3, a downstream factor of HIF-1α linked with the initiation of autophagy in *in vivo* and *in vitro* experiments. The results of *in vitro* experiments indicated the expression levels of HIF-1α, BNIP3, and LC-3II were concomitantly increased during hCG-induced luteinization of granulosa cells (Figures 3A–D), whereas transfection of siRNA targeting BNIP3 obviously reduced the expression levels of LC-3II and StAR in these granulosa-lutein cells with no significant changes of HIF-1α (Figures 3A–D). Similarly, These results of *in vivo* study revealed that inhibition of HIF-1α binding activity by echinomycin curbed the expression levels of BNIP3 and LC-3II in luteal cells (Figures 3E,F). These results clearly demonstrated that HIF-1α/BNIP3 pathway is involved in the regulation of autophagy initiation and the luteinization of granulosa cells *in vivo* and *in vitro*.

### Up-Regulated Bax Contributes to the Apoptosis of Luteal Cells During the Formation of Corpus Luteum

**Figure 4:**
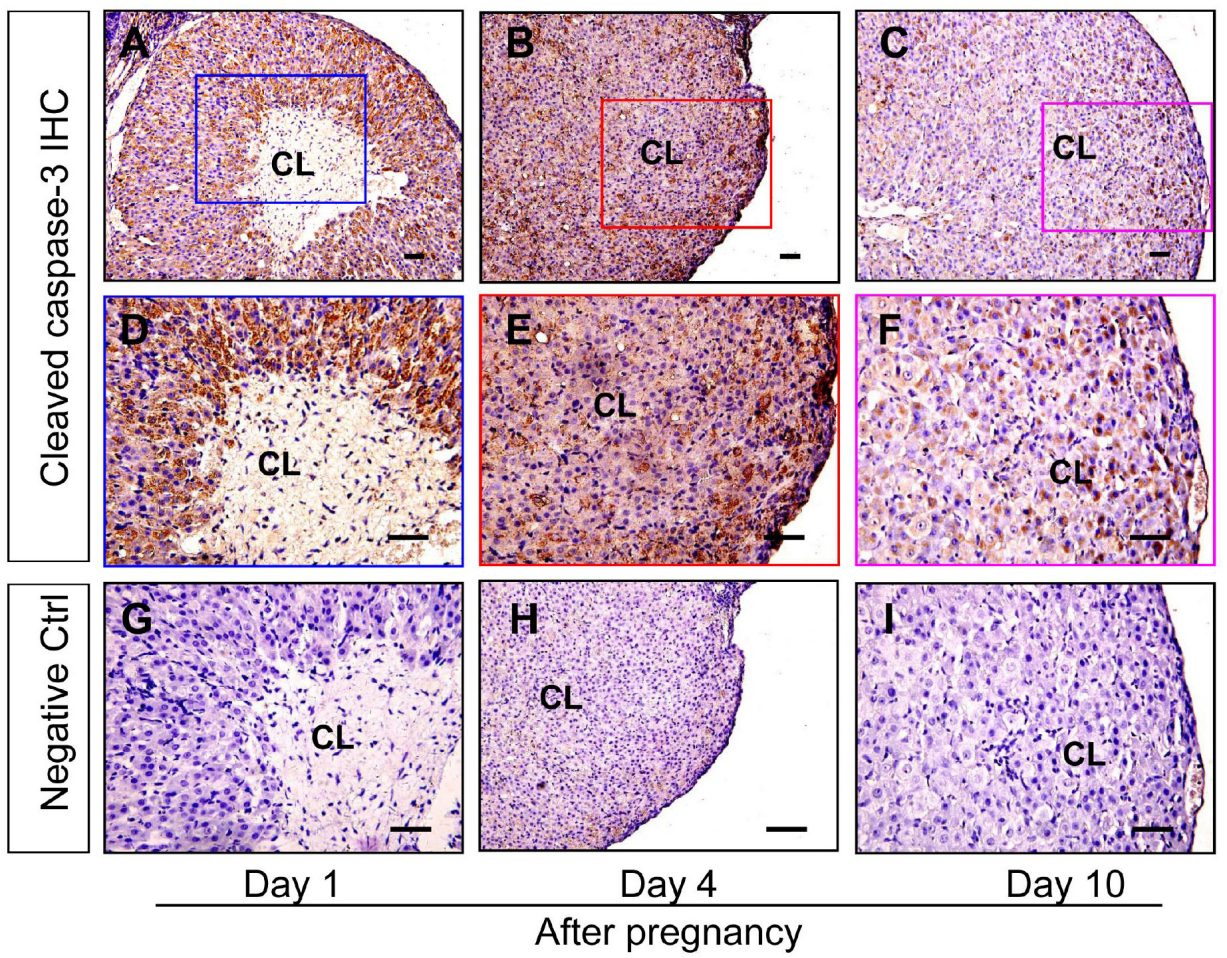
Immunohistochemical staining of cleaved caspase-3 during the development of corpus luteum. Three time points (Day 1, 4, and 10 after pregnancy) were selected and represented as the developmental process of corpus luteum according to our previous reports. Ovarian sections of each point (**A,D,G** for Day 1; **B,E**, and H for Day 4; **C,F,I** for Day 10) were immunostained for cleaved caspase-3 and counterstained with hematoxylin. The cleaved caspase-3 immunohistochemical signals appear brown, and the background counterstaining appears blue **(A–F)**. Negative control remained unstained, lacking primary antibody instead of serum **(G–I)**. CL, corpus luteum, bar = 100 μm.

**Figure 5:**
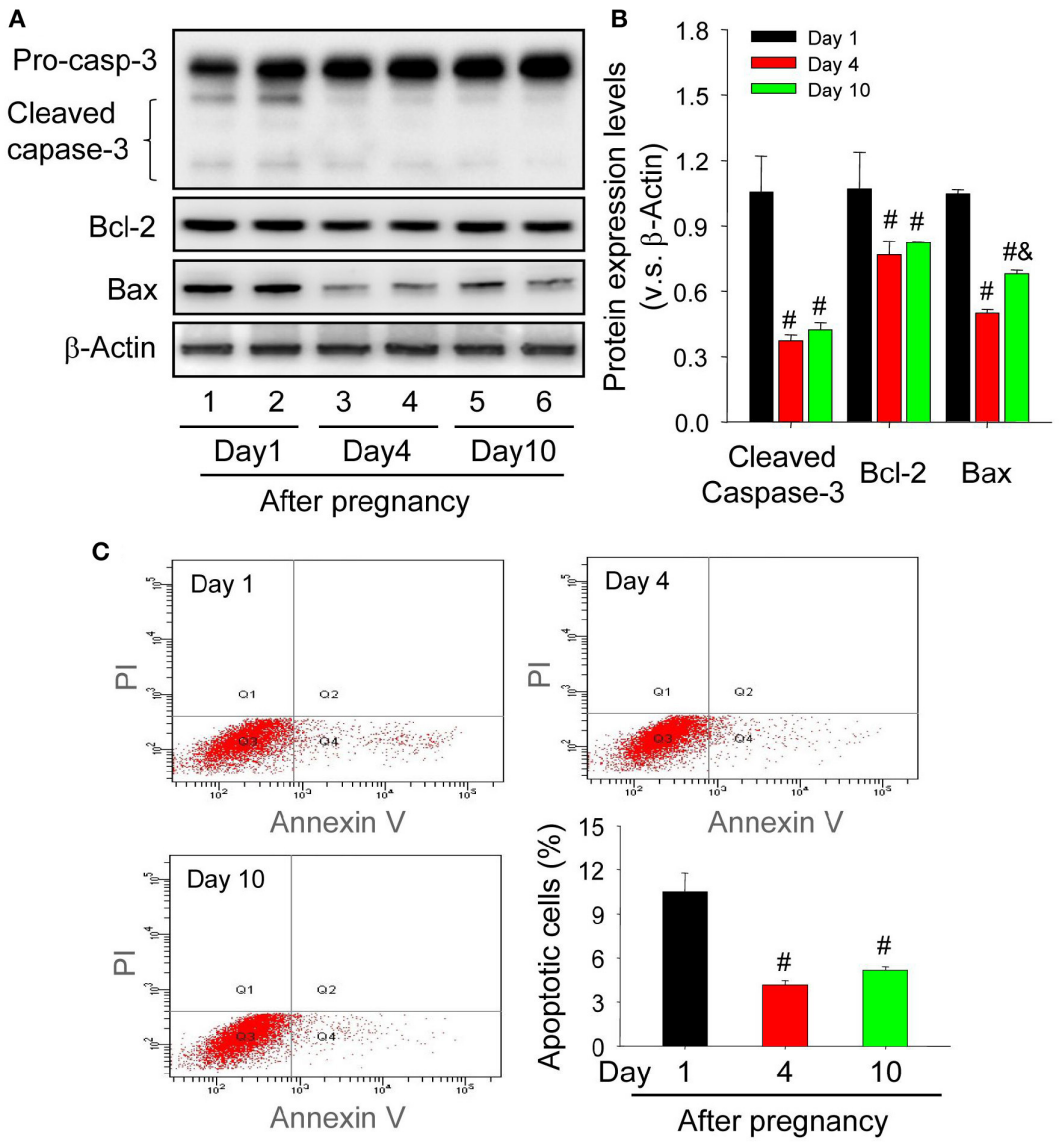
Changes of luteal cell apoptosis levels during the development of corpus luteum. **(A)** Expression changes of caspase-3, Bcl-2, and Bax proteins in luteal cells during the development of corpus luteum (from Day 1 to 10). **(B)** Summarized intensities of cleaved caspase-3, Bcl-2, and Bax bloting normalized to the control (Day 1). **(C)** The representative images of Annexin V-PI staining by flow cytometry and the quantitative analysis of luteal cell apoptosis during the development of corpus luteum. The luteal cells were obtained from the pregnant corpus luteum on Day 1, 4, and 10. Each value represents the mean ± SE. One-way analysis of variance (ANOVA) was used to analyze the data, followed by a Tukey's multiple range test. *n* = 6. ^#^*P* < 0.05, vs. the group in black. ^&^*P* < 0.05, vs. the group in red.

Granulosa cells and luteal cells are endowed with remarkable differences of cellular physiologies, including the remolding of cellular shapes and the alteration of cell metabolic mechanisms (Hsueh et al., 1984). Obviously, granulosa cells are bathed in an avascular environment, whereas luteal cells are immersed in the luxurious blood network of corpus luteum (Galvão et al., 2013), implying that granulosa cells and luteal cells may have different tolerance for hypoxia. To investigate whether hypoxia exaggerated the survival of luteal cells of the newly formed corpus luteum, the present study thus examined the expression of apoptosis related proteins. The immunohistochemical staining of cleaved caspase-3 showed that the staining intensity is the strongest in corpus luteum from the pregnant ovary on Day 1, whereas subsided on Day 4, the time when the invasion of capillary vessel is completed (Meyer and Bruce, 1980), and Day 10, the time when vascular network is fully developed and vessel volume begins to increase (Rowe et al., 2002) (Figure 4). Further analysis of Western blotting also indicated the activation of caspase-3 in the nascent corpus luteum (Day 1) compared with that of Day 4 and 10 (Figures 5A,B). Besides the luteal cells were separated and analyzed the apoptosis by flow cytometry. The luteal cells that undergo early apoptosis were stained by Annexin V-FITC kit, and the dead cells were stained by propidium iodide. The results showed that apoptotic cells of nascent corpus luteum were higher than those of Day 4 and 10 (Figure 5C), whereas no differences of dead cells could be observed between Day 1, 4, and 10 (Figure 5C). In order to explore the mechanism of caspase-3 activation, the present study also detected the expression of Bcl-2 and Bax in the pregnant corpus luteum (Figures 5A,B). The results indicated that the expression level of Bcl-2 was high during the formation of corpus luteum, while Bax expression was obviously higher than those on Day 4 and 10 after pregnancy (Figures 5A,B). These findings indicated that the activation of Bax contributed to the occurrence of luteal cell apoptosis during the formation of corpus luteum.

### Decreased Mitochondrial Potential Is Responsible for the Release of Cytochrome c in Luteal Cells During the Formation of Corpus Luteum

**Figure 6:**
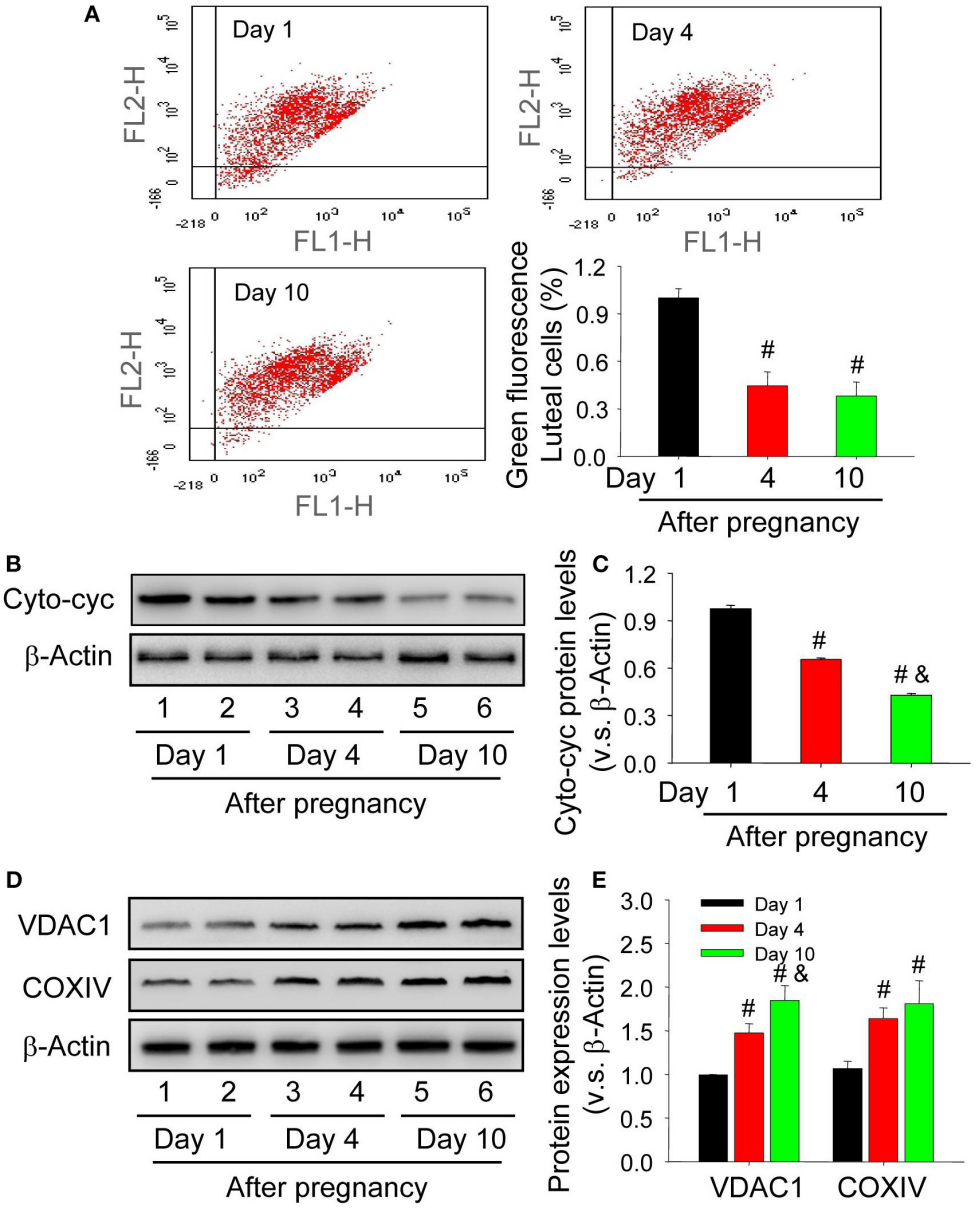
Unsteadiness of mitochondria contributes to the release of cytochrome c in the cytoplasm of luteal cells. **(A)** Mitochondrial membrane potential was evaluated by JC-1 staining and analyzed by flow cytometry. The integral segment is labeled as JC-1 red (upper right fraction) and the mitochondria with low potential is labeled as JC-1 green (damaged fraction). **(B)** Expression changes of cytoplasmic cytochrome c in luteal cells during the development of corpus luteum (from Day 1 to 10). **(C)** Summarized intensities of cytoplasmic cytochrome c bloting normalized to the control (Day 1). **(D)** Expression changes of VDAC1 and COXIV in luteal cells during the development of corpus luteum. **(E)** Summarized intensities of VDAC1 and COXIV bloting normalized to the control (Day 1). Each value represents the mean ± SE. One-way analysis of variance (ANOVA) was used to analyze the data, followed by a Tukey's multiple range test. *n* = 6. Cyto-cyc, cytoplasmic cytochrome c, ^#^*P* < 0.05, vs. the group in black; ^&^*P* < 0.05, vs. the group in red.

The maintenance of mitochondrial potential is essential for cell homeostasis, whereas the decrease of its potential contributes to the release of contents, including cytochrome c. To elucidate whether the skew of mitochondrial balance is correlated with caspase-3 activation, the present study thus examined mitochondrial potential by JC-1 staining and the results showed that mitochondrial potential of luteal cells from the nascent corpus luteum was lower than those on Day 4 and 10 after pregnancy (Figure 6A). The mitochondria were thereafter separated and then detected about the level of cytochrome c in cell cytoplasm. The results indicated that the level of cytoplasmic cytochrome c in luteal cells of neonatal corpus luteum (Day 1) was higher than those on Day 4 and 10 (Figures 6B,C), indicating the unsteadiness of mitochondrial balance in luteal cells during the formation of corpus luteum. By evaluating the expression of mitochondrial marker proteins, COXIV located on the inner mitochondrial membrane and VDAC1 located on the outer mitochondrial membrane, we found COXIV and VDAC1 were maintained at a low level in luteal cells of neonatal corpus luteum (Day 1), whereas increased on Day 4 and 10 (Figures 6D,E), indicating that the up-regulation of cytoplasm cytochrome c may not be caused by mitochondria accumulation. These findings suggested the skew of mitochondrial potential contributed to the luteal cell apoptosis by increased cytoplasmic cytochrome c during the formation of corpus luteum.

### Autophagy Is Induced in Luteal Cells During the Formation of Corpus Luteum

**Figure 7:**
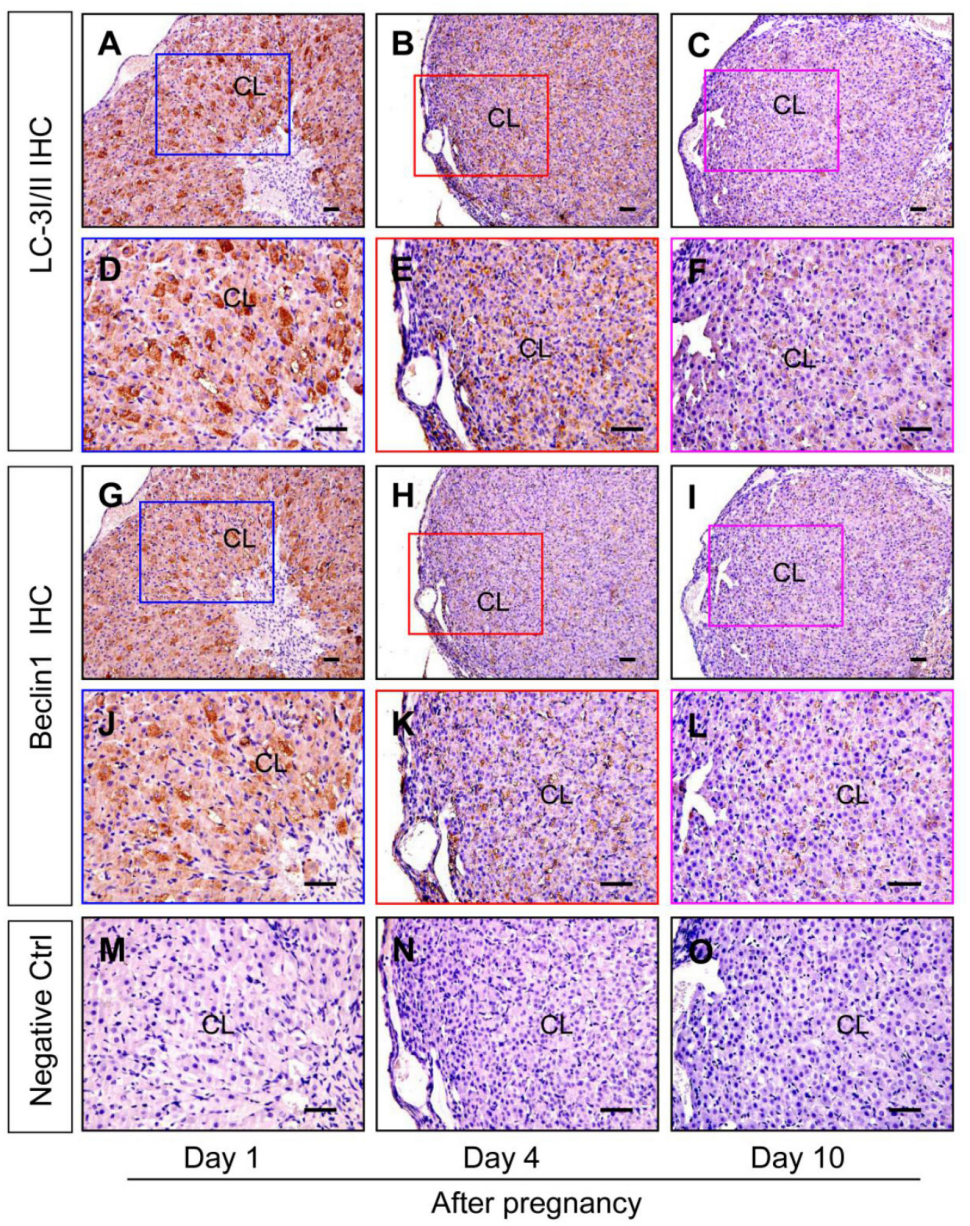
Immunohistochemical staining of beclin1/LC-3I/II during the development of corpus luteum. Three time points (Day 1, 4, and 10 after pregnancy) were selected and represented as the developmental process of corpus luteum according to our previous reports. Ovarian sections of each point (**A,D,G,J,M** for Day 1; **B,E,H,K** for Day 4; **C,F,I,L,O** for Day 10) were immunostained for beclin1/LC-3I/II and counterstained with hematoxylin. The beclin1/LC-3I/II immunohistochemical signals appear brown, and the background counterstaining appears blue (**A–F** for LC-3I/II and **G–L** for Beclin1). Negative control remained unstained, lacking primary antibody instead of serum **(M–O)**. CL, corpus luteum, bar = 100 μm.

**Figure 8:**
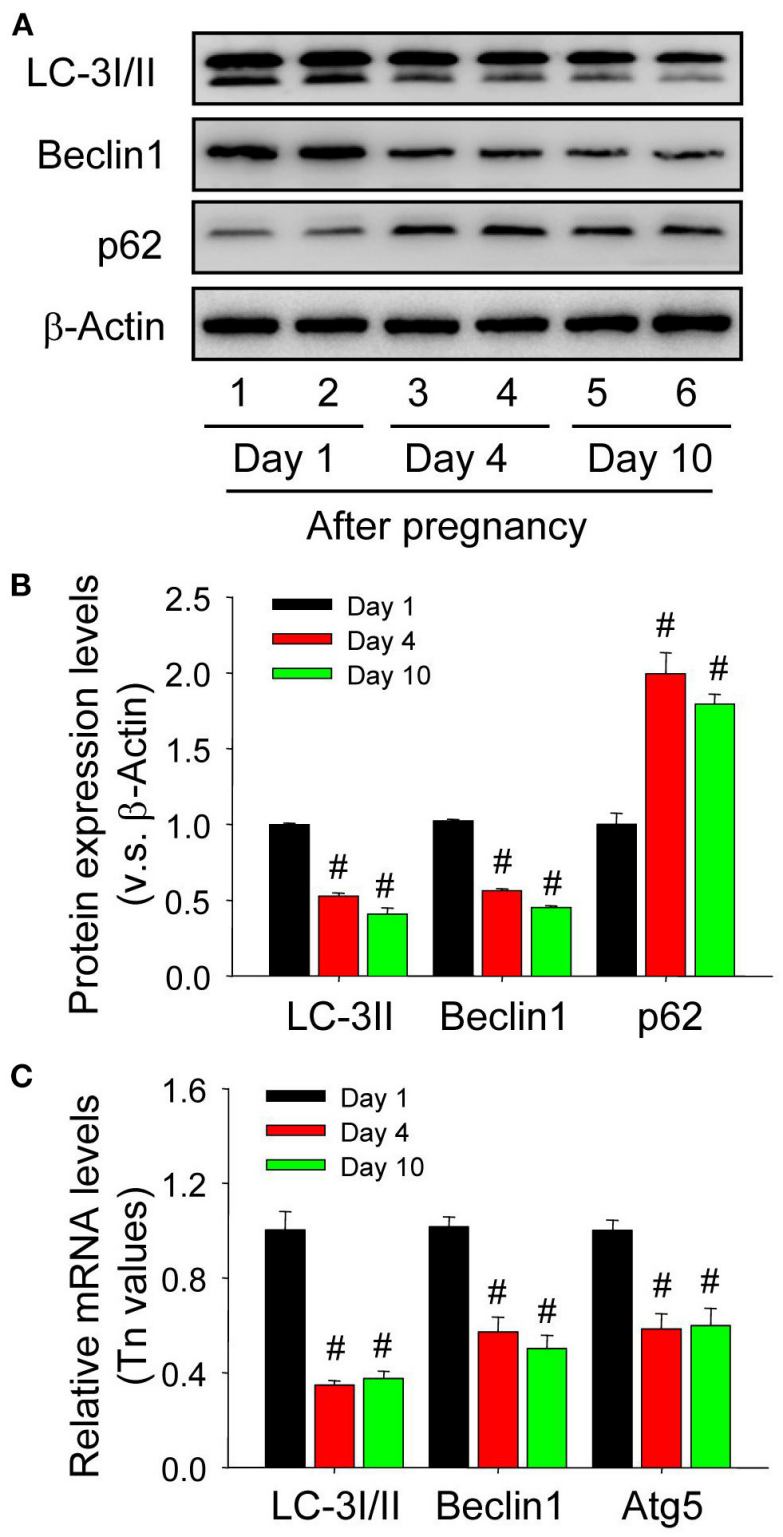
Expression changes of autophagy-related proteins in the luteal cells during the development of corpus luteum. **(A)** Expression changes of LC-3I/II, beclin1, and p62 proteins in luteal cells during the development of corpus luteum (from Day 1 to 10). **(B)** Summarized intensities of LC-3I/II, beclin1, and p62 bloting normalized to the control (Day 1). **(C)** Expression Changes of LC-3I/II, beclin1, and Atg5 mRNA in the luteal cells during the development of corpus luteum. The luteal cells were obtained from the pregnant corpus luteum on Day 1, 4, and 10. Each value represents the mean ± SE. One-way analysis of variance (ANOVA) was used to analyze the data, followed by a Tukey's multiple range test. *n* = 6. ^#^*P* < 0.05, vs. the group in black.

Although autophagy is involved in the luteinization of granulosa cells after ovulation, the expression changes of autophagy during the formation of corpus luteum still remains unknown. The present study thereafter examined the expression of autophagic marker proteins, LC-3I/II, beclin1, and p62. The results of immunohistochemistry showed ovarian staining intensities of LC-3I/II and beclin1 on Day 1 were much stronger than those on Day 4 and 10 (Figure 7), which was further confirmed by Western blotting results of LC-3II and beclin1 (Figures 8A,B), besides the opposite tendency of p62 (Figures 8A,B). In additional, ovairan mRNA levels of LC-3I/II, beclin1, and Atg5 on Day 1 were also higher than those on Day 4 and 10 (Figure 8C). Inhibition of autophagy flux by chloroquine obviously increased the apoptosis of granulosa-lutein cells *in vivo* (Supplementary Figure 2). These results suggested that autophagy was obviously induced during the luteinization of granulosa cells after ovulation and contributed to the formation of corpus luteum.

### HIF-1α/BNIP3 Is Involved in the Induction of Autophagy During the Formation of Corpus Luteum

**Figure 9:**
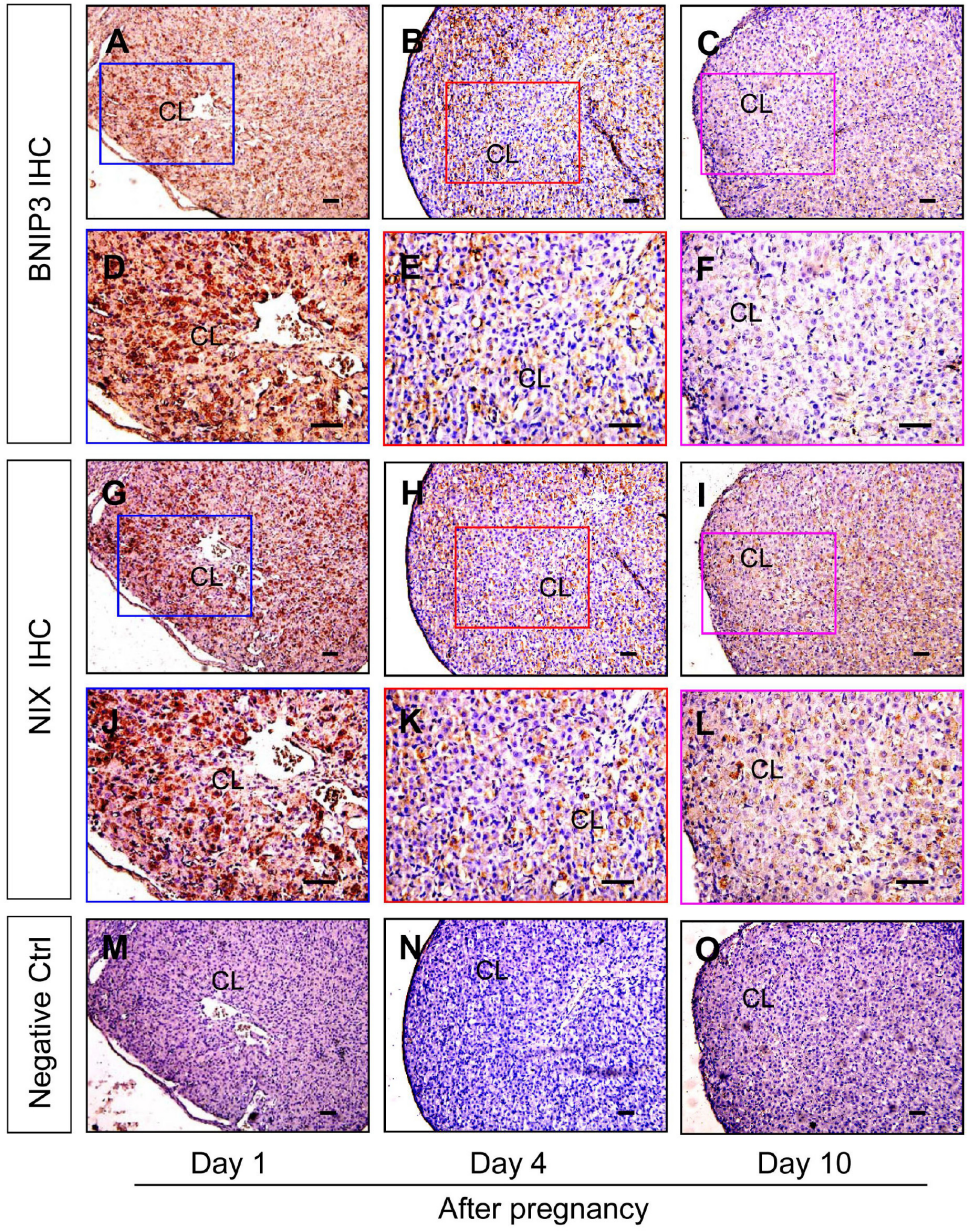
Immunohistochemical staining of BNIP3/NIX during the development of corpus luteum. Three time points (Day 1, 4, and 10 after pregnancy) were selected and represented as the developmental process of corpus luteum according to our previous reports. Ovarian sections of each point (**A,D,G,J,M** for Day 1; **B,E,H,K,N** for Day 4; **C,F,I,L,O** for Day 10) were immunostained for BNIP3/NIX and counterstained with hematoxylin. The BNIP3/NIX immunohistochemical signals appear brown, and the background counterstaining appears blue (**A–F** for BNIP3 and **G–L** for NIX). Negative control remained unstained, lacking primary antibody instead of serum **(M–O)**. CL, corpus luteum, bar = 100 μm.

**Figure 10:**
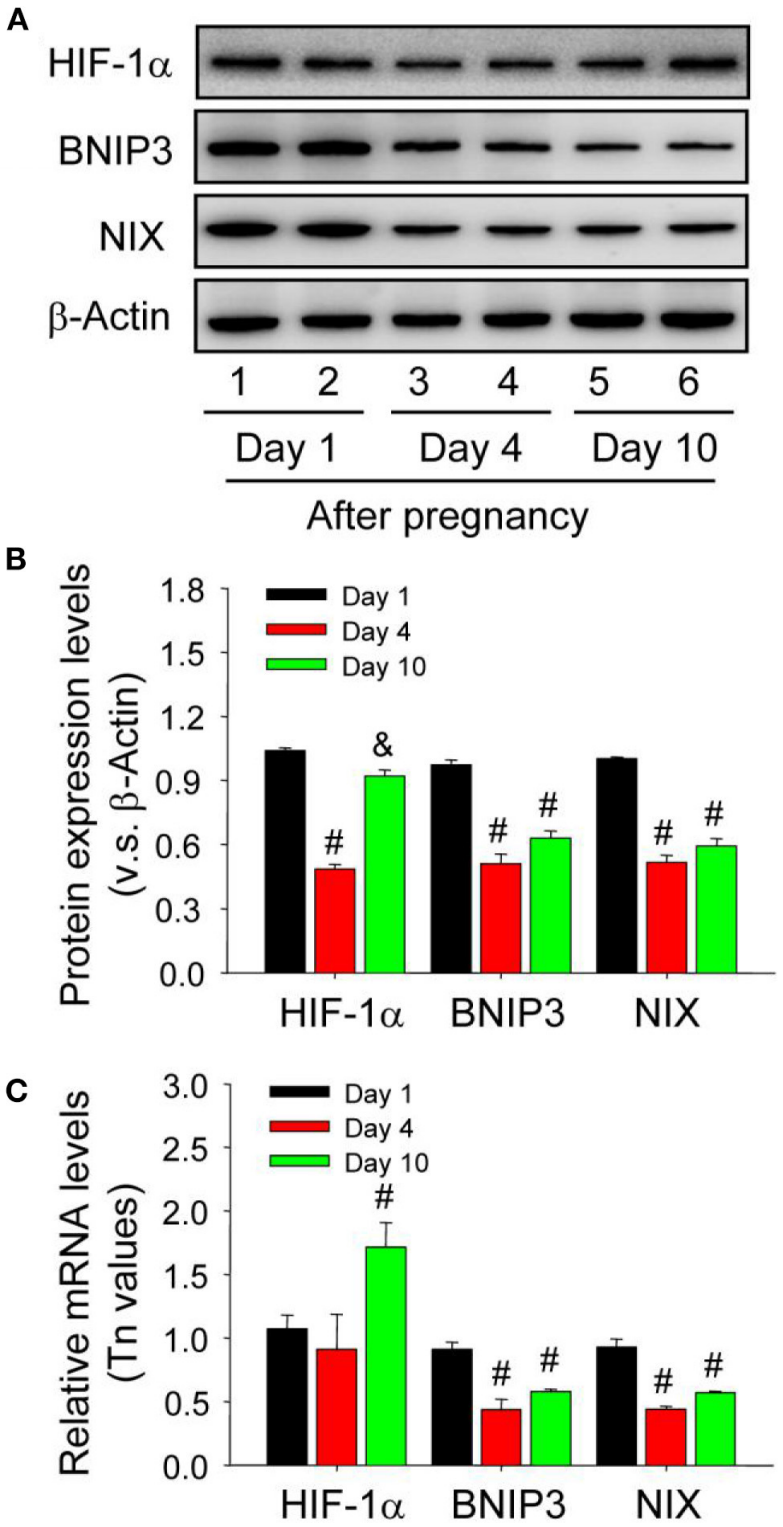
Expression changes of HIF-1α/BNIP3/NIX proteins in the luteal cells during the development of corpus luteum. **(A)** Expression changes of HIF-1α, BNIP3, and NIX proteins in luteal cells during the development of corpus luteum (from Day 1 to 10). **(B)** Summarized intensities of HIF-1α, BNIP3, and NIX bloting normalized to the control (Day 1). **(C)** Expression changes of HIF-1α, BNIP3, and NIX mRNA in the luteal cells during the development of corpus luteum. The luteal cells were obtained from the pregnant corpus luteum on Day 1, 4, and 10. Each value represents the mean ± SE. One-way analysis of variance (ANOVA) was used to analyze the data, followed by a Tukey's multiple range test. *n* = 6. ^#^*P* < 0.05, vs. the group in black. ^&^*P* < 0.05, vs. the group in red.

Neonatal corpus luteum undergoes a period of non-vascular term caused by the hysteresis of vascular invasion (Niswender et al., 2000; Robinson et al., 2009; Galvão et al., 2013) and our previous studies also revealed the important regualtion of HIF-1α signaling during the luteral development (Wu et al., 2015). Given the essential roles of BNIP3/NIX in HIF-1α-mediated autophagy under hypoxia, the present study thus examined their expression changes during the formation of corpus luteum by immunohistochemistry and found BNIP3/NIX staining in ovarian luteal cells on Day 1 was stronger than those on Day 4 and 10 (Figure 9), which was further confirmed by Western blotting results of BNIP3/NIX (Figures 10A,B) and real-time PCR results of BNIP3/NIX (Figure 10C). Interestingly, ovarian expression level of HIF-1α protein on Day 1 was significantly higher than that on Day 4 (Figure 10B), while no obvious change of HIF-1α mRNA expression was found between the ovaries on Day 1 and 4 (Figure 10C). These results suggested HIF-1α was induced by hypoxia after ovulation, which activated BNIP3/NIX for the induction of autophagy during the formation of corpus luteum.

### BNIP3 Disrupts Bcl-2/Beclin1 Complex and Induces Beclin1-Dependent Autophagy

**Figure 11:**
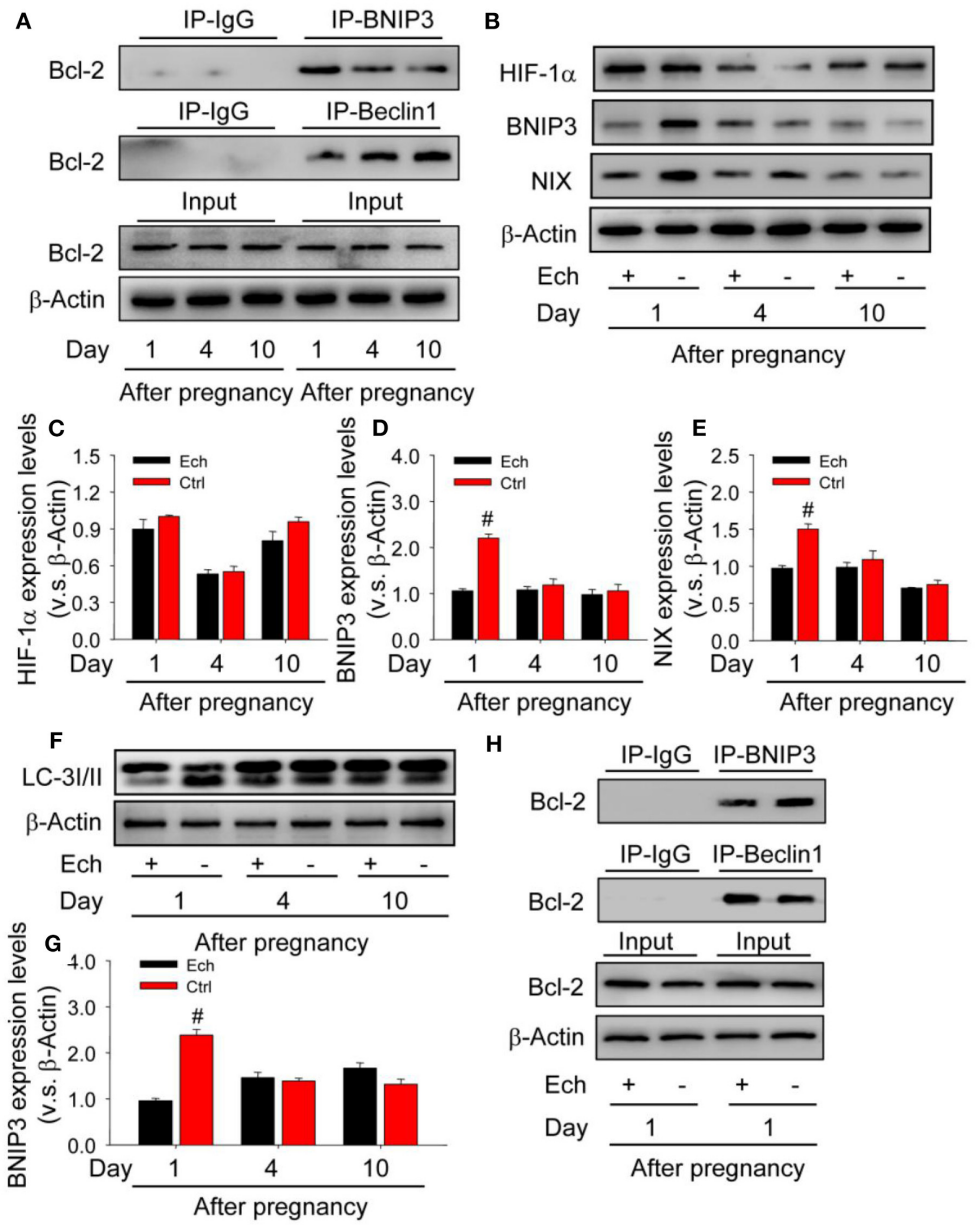
Competitive binding of BNIP3/beclin1 with bcl-2 contributes to the induction of autophagy during the formation of corpus luteum. **(A)** Expression changes of Bcl-2 in the corpus luteum obtained from the pregnant ovary and homogenized for co-immunoprecipitation with anti-BNIP3 antibody or anti-beclin1 antibody during the development of corpus luteum. **(B)** Expression changes of HIF-1α, BNIP3, and NIX proteins in luteal cells during the development of corpus luteum with echinomycin treatment. **(C)** Summarized intensities of HIF-1α blotting normalized to the control (Day 1 with echinomycin treatment). **(D)** Summarized intensities of BNIP3 blotting normalized to the control. **(E)** Summarized intensities of NIX blotting normalized to the control. **(F)** Expression changes of LC-3I/II protein in luteal cells during the development of corpus luteum with echinomycin treatment. **(G)** Summarized intensities of LC-3I/II bloting normalized to the control. **(H)** Expression changes of Bcl-2 in the corpus luteum obtained from the pregnant ovary and homogenized for co-immunoprecipitation with anti-BNIP3 antibody or anti-beclin1 antibody during the formation of corpus luteum (Day 1 after pregnancy). Each value represents the mean ± SE. One-way analysis of variance (ANOVA) was used to analyze the data, followed by a Tukey's multiple range test. *n* = 6. Ech, echinomycin, ^#^*P* < 0.05, vs. the group in black.

As shown that HIF-1α/BNIP3 is activated during the induction of autophagy, but the regulatory mechanism during the formation of corpus luteum needs further examination. Given the importantly regulatory role of Bcl-2 in the induction of autophagy, the present study therefore detected the levels of Bcl-2/beclin1 and Bcl-2/BNIP3 complex by co-Immunoprecipitation (Co-IP) during the formation of corpus luteum (Figure 11A) and then found the level of Bcl-2/BNIP3 complex on Day 1 was much higher than those on Day 4 and 10, while the level of Bcl-2/beclin1 complex with opposite tendency (Figure 11A), indicating beclin1 was released from Bcl-2/beclin1 complex and then induced autophagy.

To further confirm the regulatory role of HIF-1α/BNIP3-mediated autophagy during the formation of corpus luteum, the present study thus examined the expression changes of autophagy after echinomycin treatment (Figures 11B–F) and then found BNIP3/NIX (Figures 11D,E) and LC-3II (Figure 11G) expression decreased without HIF-1α protein expression changed (Figure 11C), indicating the regulatory role of HIF-1α/BNIP3 in the induction of autophagy. Further Co-IP examination found Bcl-2/BNIP3 complex increased and Bcl-2/beclin1 complex decreased in the ovaries with echinomycin treatment on Day 1 after pregnancy (Figure 11H). These results together suggested HIF-1α induced the expression of BNIP3, which disrupted pre-existing Bcl-2/beclin1 complexes through competing bind with Bcl-2 for the formation of Bcl-2/BNIP3 complexes. And then released beclin1 activated the initiation of autophagy during the formation of corpus luteum.

### HIF-1α-Mediated Autophagy Contributes to the Survival of Luteinizing Granulosa Cells by Eliminating Excessive Mitochondria

**Figure 12:**
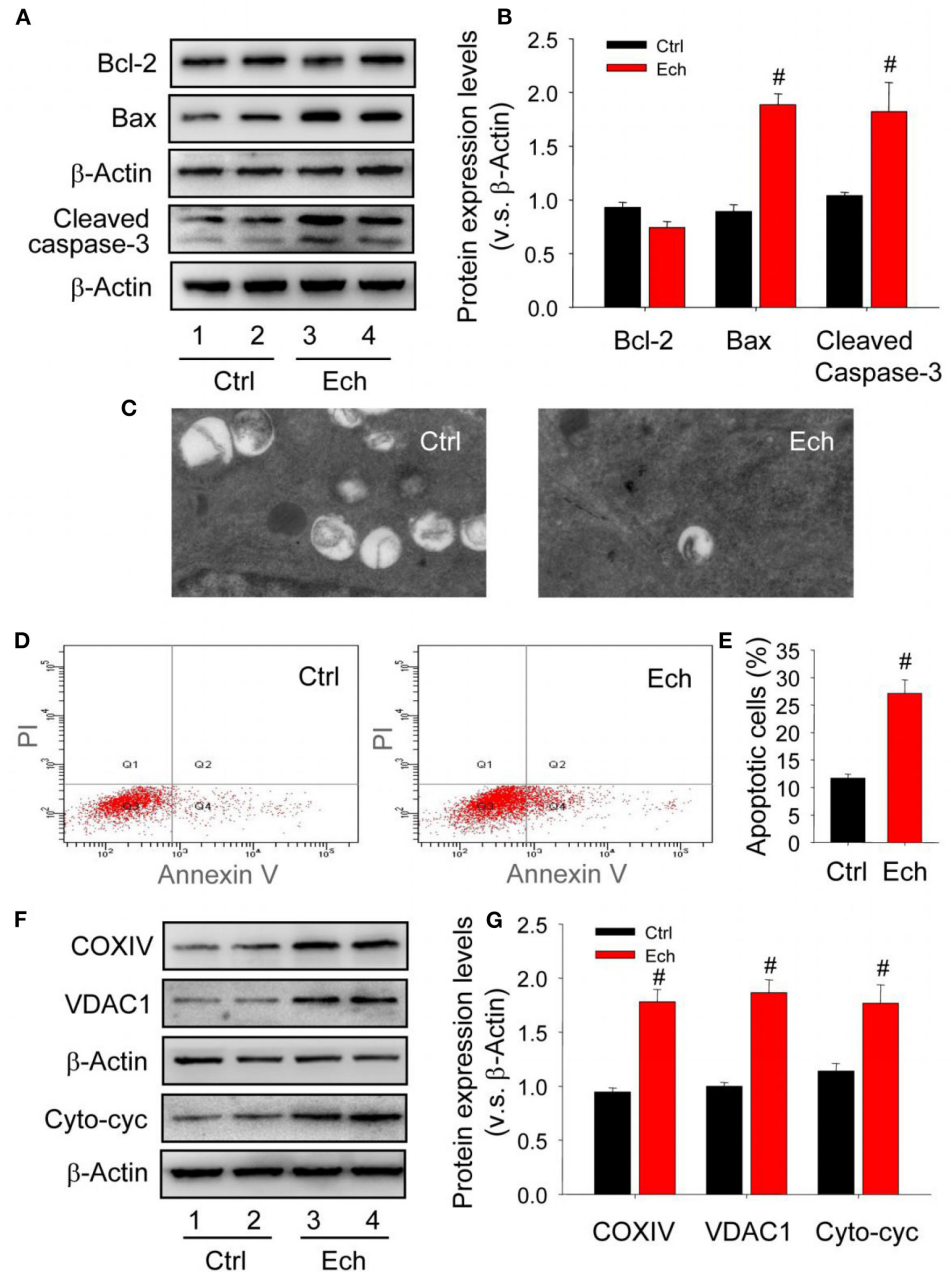
Mitochondrial impairment contributes to the apoptosis during the formation of corpus luteum. **(A)** Expression changes of Bcl-2, Bax, and cleaved caspase-3 during the formation of corpus luteum after echinomycin treatment. **(B)** Summarized intensities of Bcl-2, Bax, and cleaved caspase-3 blotting normalized to the control. **(C)** TEM images of the autophagosome in luteal cells with or without echinomycin treatment. **(D)** Representative images of Annexin V-PI staining in luteal cells obtained from pregnant ovary with or without echinomycin treatment. **(E)** Quantitative analysis of luteal cell apoptosis by flow cytometry during the formation of corpus luteum with or without echinomycin treatment. **(F)** Expression changes of VDAC1, COXIV, and cytoplasmic cytochrome c in luteal cells during the formation of corpus luteum (Day 1) with or without echinomycin treatment. **(G)** Summarized intensities of VDAC1, COXIV, and cytoplasmic cytochrome c bloting normalized to the control. Each value represents the mean ± SE. One-way analysis of variance (ANOVA) was used to analyze the data, followed by a Tukey's multiple range test. *n* = 6. Ctrl, control; Cyto-cyc, cytoplasmic cytochrome c, ^#^*P* < 0.05, vs. the group in black.

In mammalian cells, the induction of autophagy is highly associated with the survival or death of cells (Zhu et al., 2010). The reason why corpus luteum can be successively developed with high apoptosis levels during the formation of corpus luteum remains unclearly. Given the important role of HIF-1α during the luteal development, the present study examined the changes of luteal cell apoptosis and cytoplasmic mitochondria after echinomycin treatment, and then found Bcl-2 expression was maintained accompanied by increased Bax expression and activated caspase-3 in the forming corpus luteum after echinomycin treatment (Figures 12A,B). The results of TEM demonstrated echinomycin inhibited the autophagy during the formation of corpus luteum (Figure 12C, Supplementary Figure 3). In additional, luteal cells were separated and the apoptotic levels were examined by annexin V-PI staining kit, which indicated echinomycin enhanced the apoptotic level in luteinizing granulosa cells (Figures 12D,E). Notably, the present study also found echinomycin led to the accumulation of mitochondria during the formation of corpus luteum as evidenced by up-regulated COXIV and VDAC1 expressions (Figures 12F,G). Further analysis demonstrated the levels of cytoplasmic cytochrome c increased during the formation of corpus luteum (Figures 12F,G). Thus, the elimination of mitochondria by HIF-1α-medated autophagy may be a self-protective mechanism to alleviate the activation of caspase-3 caused by mitochondrial cytochrome c release.

## Discussion

In mammal, the timely formation of capillary vessel network is required for the transportation of nutrition and oxygen to maintain the survival and development of tissue cells (Shan et al., 2004), whereas the hysteresis of capillary vessel invasion is tightly related to tissue hypoxia. Owing to the particularity of physiological structures, the granulosa cells and thereafter differentiating luteal cells are surrounded by an avascular environment and immersed in a hypoxia environment (Niswender et al., 2000). HIF-1 is a helix-loop-helix transcriptional factor consists of HIF-1α and HIF-1β, the transcriptional activation of which is linked with many oxygen-sensitive genes that deeply involved in various developmental and physiological processes (Semenza, 2000). It has been established that HIF-1α is induced with the decrease of O_2_ concentration in tissue or cells, and the activation of HIF-1α signaling pathway is generally launched as a retrenchment strategy available for many cell types when suffered to oxygen insufficient, whose activity is accordingly responsible for the maintenance of metabolic homeostasis and the survival of cells that immersed in unfavorable niches (Semenza, 1999; Vaupel, 2004). Intriguingly, HIF-1α is also recognized as one of the factors that involved in hormonal regulation during the differentiation and luteinization of follicular cells in mice (Tam et al., 2010). Inhibition of HIF-1α blocks the ovulation and luteinizationin mice (Kim et al., 2009; Chen et al., 2017), indicating the vital roles of HIF-1α signaling in female ovarian functions. By *in vivo* and *in vitro* experiments, the present study indicated that HIF-1α was significantly induced during the luteinization of granulosa cells, and HIF-1α specific inhibitor echinomycin encumbered the differentiation of granulosa cells during the formation of corpus luteum through inhibiting HIF-1α activity, suggesting an essential role for HIF-1α during the luteinization of granulosa cells and the formation of corpus luteum. However, the mechanism of HIF-1α regulating the luteinization of granulosa cells after ovulation still remains unknown.

Until recent years, the researches of autophagy thread a light on this issue (Chen et al., 2008; Mazure and Pouysségur, 2010; Hsieh et al., 2015; Shen et al., 2017; Tang et al., 2017b). Autophagy is an evolutionarily conserved cellular catabolic mechanism, the induction of which is highly associated with various cellular physiologies (Chen et al., 2008; Hsieh et al., 2015). Under hypoxia, up-regulation of HIF-1α can invoke autophagic mechanism to maintain the balance of cell metabolism (Mazure and Pouysségur, 2010). Previously, evidences also revealed that HIF-1α-induced autophagy plays a positive role in the survival of granulosa cells by protecting them from oxidative stress or exempting them from damages induced by chemical reagents (Shen et al., 2017; Tang et al., 2017b). These evidences stick out the importance of HIF-1α-mediated autophagy in the functions of granulosa cells. Although the relationship between HIF-1α and granulosa cell differentiation has been reported previously (Chen et al., 2017; Fadhillah et al., 2017; Zhou et al., 2018; Yadav et al., 2019), the underlying mechanism still remain largely unknown. Here, the present study has demonstrated that HIF-1α was exerted in luteinizing granulosa cells through mediating the expressions of autophagy, as the induce of autophagy was found during the formation of corpus luteum, and inhibition of HIF-1α activity by echinomycin contributed to decreased autophagy. Moreover, the present study utilized 3-MA to inhibit autophagy during this process and found the essential role of autophagy in the luteinization of granulosa cells during the formation of corpus luteum

Interestingly, BNIP3 and NIX are two downstream homologous target proteins of transcription factor HIF-1α, which are highly associated with the induction of HIF-1α-mediated autophagy under hypoxia conditions (Mazure and Pouysségur, 2009). Available evidences have also indicated the regulation of HIF-1α on BNIP3 expression in mouse granulosa cells (Zhou et al., 2017). Therefore, the present study examined whether BNIP3 was involved in the induction of autophagy during the luteinizaiton of granulosa cells and found knockdown BNIP3 by siRNA *in vitro* inhibited the induce of autophagy and the differentiation of granulosa cells regardless of up-regulated HIF-1α, while down-regulated BNIP3 by HIF-1α specific inhibitor echinomycin *in vivo* also decreased the level of autophagy. These results suggested HIF-1α-mediated autophagy is regulated in a BNIP3-dependent manner, which is consistent with the mechanism of hypoxia-induced osteoclastogenesis revealed by Zhao *et al*. (Zhao et al., 2012). Besides the evidences indicated that HIF-1α/NIX-mediated mitophagy plays an essential role in the reconfiguration of retinal ganglion cell metabolism during the neurogenesis (Esteban-Martínez and Boya, 2018). Combined with our findings, it is possible that HIF-1α/BNIP3-mediated autophagy is tightly related with the rearrangement of cellular mitochondria distribution during mammalian cell differentiation under hypoxia.

Although corpus luteum is evolved from ovulated follicles, it is unlike follicles and endowed with one of the highest blood flow rates in the body to ensure the sufficient oxygen and nutrition supply for hormonal synthesizing (Bruce and Moor, 1976). The formation of this luxurious blood vessel network could be achieved within a few days after ovulation, coinciding with the luteal functions. It has been demonstrated that newly formed blood vessels invade those predetermined luteal structure from the theca during the dissolution of membrane granulosa cells and march toward the center of the ruptured follicle, whereas the complete of vessels invade lasts for a few days according to the specific schedule. Thus, within a given luteal phase the cells within neonatal luteal tissue are also suffered from low oxygen supply analogous to that observed in granulosa cells (Amselgruber et al., 1999). Interestingly, the present study observed the higher levels of Bax, cleaved caspase-3 and cytoplasmic cytochrome c in neonatal corpus luteum, indicating an arise of the apoptosis of luteinizing granulosa cells, which may be caused by the inflammatory response after ovulation (Espey, 1994) and the avascular environment with the vascular invasion lapse (Mazure and Pouysségur, 2009; Fadhillah et al., 2017).

Notably, it has indicated that HIF-1α-mediated autophagy plays a pivotal role in reprograming cellular metabolism, so as to fundamentally change the source of energy supplement from oxidative phosphorylation to glycolysis under hypoxia (Zhou et al., 2018). And the failure of this transformation may partially contribute to the apoptosis of cells. In the present study, it is interesting that the mitochondria amount in the luteinizing granulosa cells on Day 1 was actually lower than those on Day 4 and 10, and inhibition of HIF-1α further promoted the activation of Bax, caspse-3, and cytoplasmic cytochrome c in these cells. These findings highlighted the contribution of HIF-1α-mediated autophagy to mitochondrial elimination during the formation of corpus luteum. The operation of such well-designed mechanism is meaningful to avoid the further release of cytochrome C, and also merit, at least partially, to the decrease of energy consumption or the reprogramming of cell metabolism under hypoxia. Work from others revealed that autophagic response is also obviously induced in pregnant uterus on Day 1 and 2 and thereafter subsides around the time of implantation (Choi et al., 2014), which is consistent with the schedule of autophagic variation revealed in present study. Similar phenomenon was also observed in trophoblasts under low oxygen (de Andrade Ramos and Witkin, 2016). Thus, autophagic response may be an essential adaptive mechanism in mammalian reproductive system, whose level could be significantly induced by extra- or intra-cellular megaevolution of niche environment. In the newly formed corpus luteum, the drastic change of intracavitary environment might be the cause of autophagic induction for inner cells.

Generally, HIF-1α/BNIP3 pathway could be efficiently mobilized in mammalian cells under hypoxia (Mazure and Pouysségur, 2009). Our results demonstrated the high expression levels of HIF-1α, BNIP3 and NIX in corpus luteum on Day 1 compared with that of Day 4 or 10, which is consistent with previous investigations observed in cattle luteal formation (Nishimura et al., 2008). Interestingly, although the expression level of HIF-1α on Day 10 was also maintained at a high level, but we did not observe the concomitant mRNA up-regulation of BNIP3 or its homologue NIX, implying that HIF-1α-medatied autophagy may not be involved during this luteal phase. Echinomycin treatment verified the role of HIF-1α in the induction of autophagy during the formation of corpus luteum. The results of Co-IP analysis further found the level of Bcl-2/BNIP3 complex was obviously higher than that of Bcl-2/beclin1 complex in the ovaries of Day 1, whereas reversed on Day 4 or 10 after pregnancy, revealing that Bcl-2 is an effective rheostat to orchestrate the induction or the inhibition of Beclin-1-dependent autophagy by forming Bcl-2/beclin1 complex (Bellot et al., 2009). However, as a member of the BH3-only subfamily proteins, BNIP3 can also participate in the regulation of autophagy *via* interacting with Bcl-2 especially under hypoxia (Mazure and Pouysségur, 2009). Our present study also demonstrated that HIF-1α-mediated up-regulation of BNIP3 can competitively binds with Bcl-2 and thereafter disrupting beclin1 from Bcl-2/beclin1 complex for the induction of autophagy during the formation of corpus luteum. Therefore, the expression of BNIP3 is an important factor influencing the differentiation of granulosa cells, while relevant investigation lags far behind. Combine with the alteration of mitochondrial status after HIF-1α inhibition, we suggest that BNIP3 may exerts its physiological role in the differentiation of granulosa cells by regulating mitochondrial functions and more evidences are required to support this speculation.

Indeed, HIF-1α has broad target genes that involved in the regulation of cell proliferation, glycolysis and inflammation. However, the expression and role of these target genes on luteinization are poorly understood. It has been demonstrated the HIF-1α can also drive the process of luteinization by enhancing the expression of vascular endothelial growth factor (VEGF), which is a potent agent required for angiogenesis. VEGF expression is obviously increased during luteinization and exert an essential role during luteal development (Tam et al., 2010; Zhang et al., 2011a,b, 2015, 2019; Wu et al., 2015; Tang et al., 2017a). In addition, HIF-1α may also affect the expression of fibroblast growth factor 2 (FGF2) during luteinization, as FGF2 level reaches its peak during luteinization which strikingly overlapping the expression of HIF-1α (Robinson et al., 2007; Tang et al., 2020). Although the role of HIF-1α during luteinization has been demonstrated for many years while its regulatory mechanism still remain to be deciphered.

**Figure 13:**
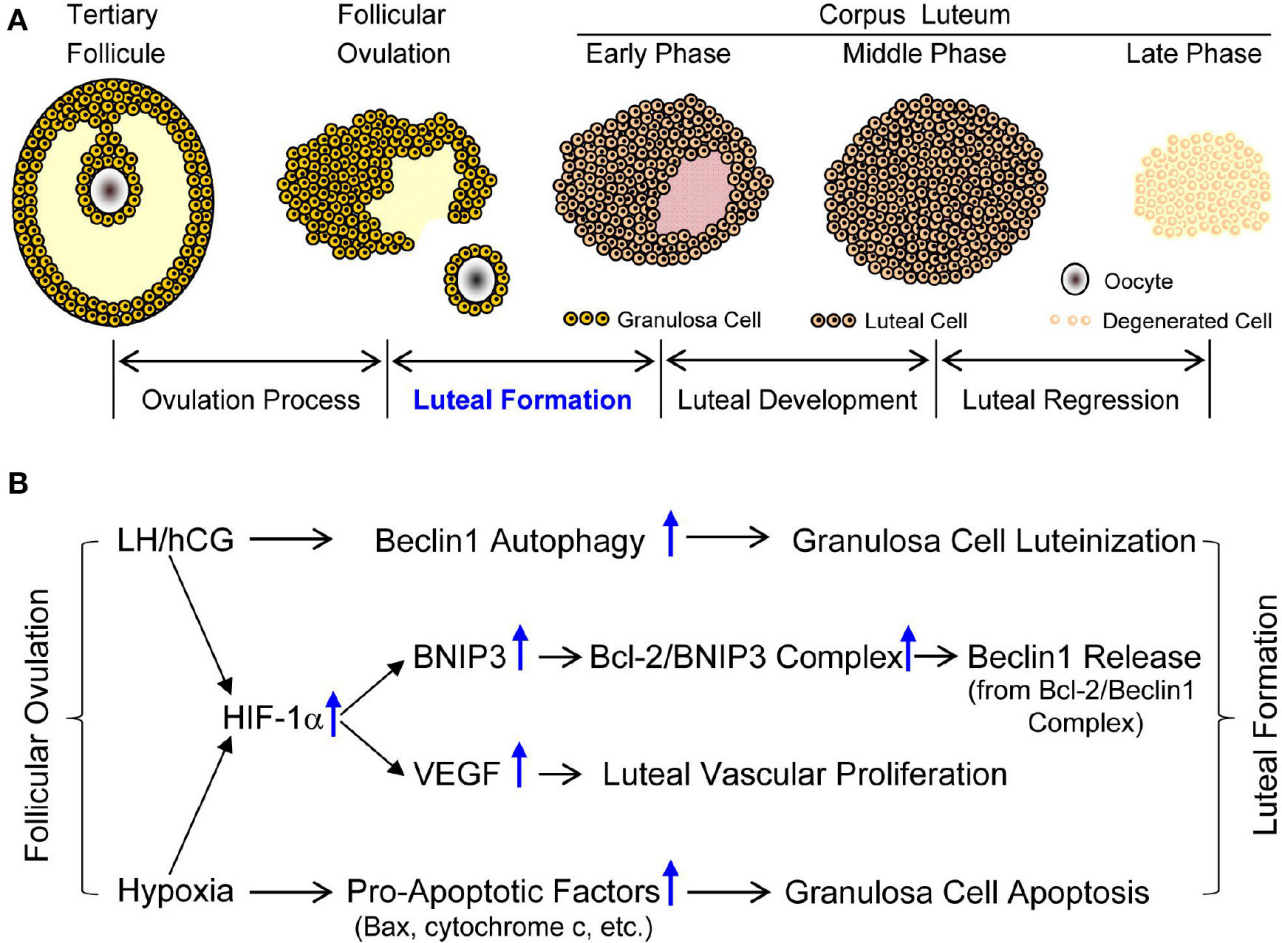
Developmental phases of corpus luteum and possible mechanism of luteal formation. **(A)** Developmental phases of corpus luteum. **(B)** Possible mechanism of luteal formation.

In summary, the present study firstly demonstrated hypoxia-induced up-regulation of HIF-1α is involved in the luteinization of granulosa cells through inducing the initiation of autophagy in a BNIP3-dependent manner (Figure 13). Furthermore, HIF-1α/BNIP3-mediated autophagy plays a positive role in the formation and establishment of corpus luteum by *in vivo* and *in vitro* experiments (Figure 13). To our knowledge, the present study firstly clarified that HIF-1α/BNIP3-mediated autophagy contributes to the luteinization of granulosa cells during the formation of pregnant corpus luteum, which will help us further understanding the luteal biology and provide us new clues for the treatment of luteal insufficiency.

## Data Availability Statement

The original contributions presented in the study are included in the article/Supplementary Material, further inquiries can be directed to the corresponding author/s.

## Ethics Statement

The animal study was reviewed and approved by the Ethics Committee on Animal Experimentation of Fujian Normal University.

## Author Contributions

The work was conceived and manuscript writing was performed by ZT, ZZ, QL, RX, HY, and ZW. The experiment was performed, the data was analyzed, and the reagents, materials, analysis tools were contributed by ZT, ZZ, QL, RX, JC, YW, YZ, YT, CS, and YL. All authors reviewed and approved the manuscript.

## Conflict of Interest

The authors declare that the research was conducted in the absence of any commercial or financial relationships that could be construed as a potential conflict of interest.
