# Supplementary material for: HIF-1α/BNIP3-Mediated Autophagy Contributes to the Luteinization of Granulosa Cells During the Formation of Corpus Luteum
Source: Front Cell Dev Biol. 2021 Jan 18;8:619924. doi: 10.3389/fcell.2020.619924 (PMC7848109; doi:10.3389/fcell.2020.619924)
Supplement: Supplementary file 1 [file Table_1.DOC]

**HIF-1α/BNIP3-Mediated Autophagy Contributes to the Luteinization of Granulosa Cells during the Formation of Corpus Luteum**

Zonghao Tang, Zhenghong Zhang, Qingqiang Lin, Renfeng Xu, Jiajie Chen, Yuhua Wang, Yan Zhang, Yedong Tang, Congjian Shi, Yiping Liu, Hongqin Yang, Zhengchao Wang

**1 Supplementary information**

**1.1 Supplementary Figure S1**


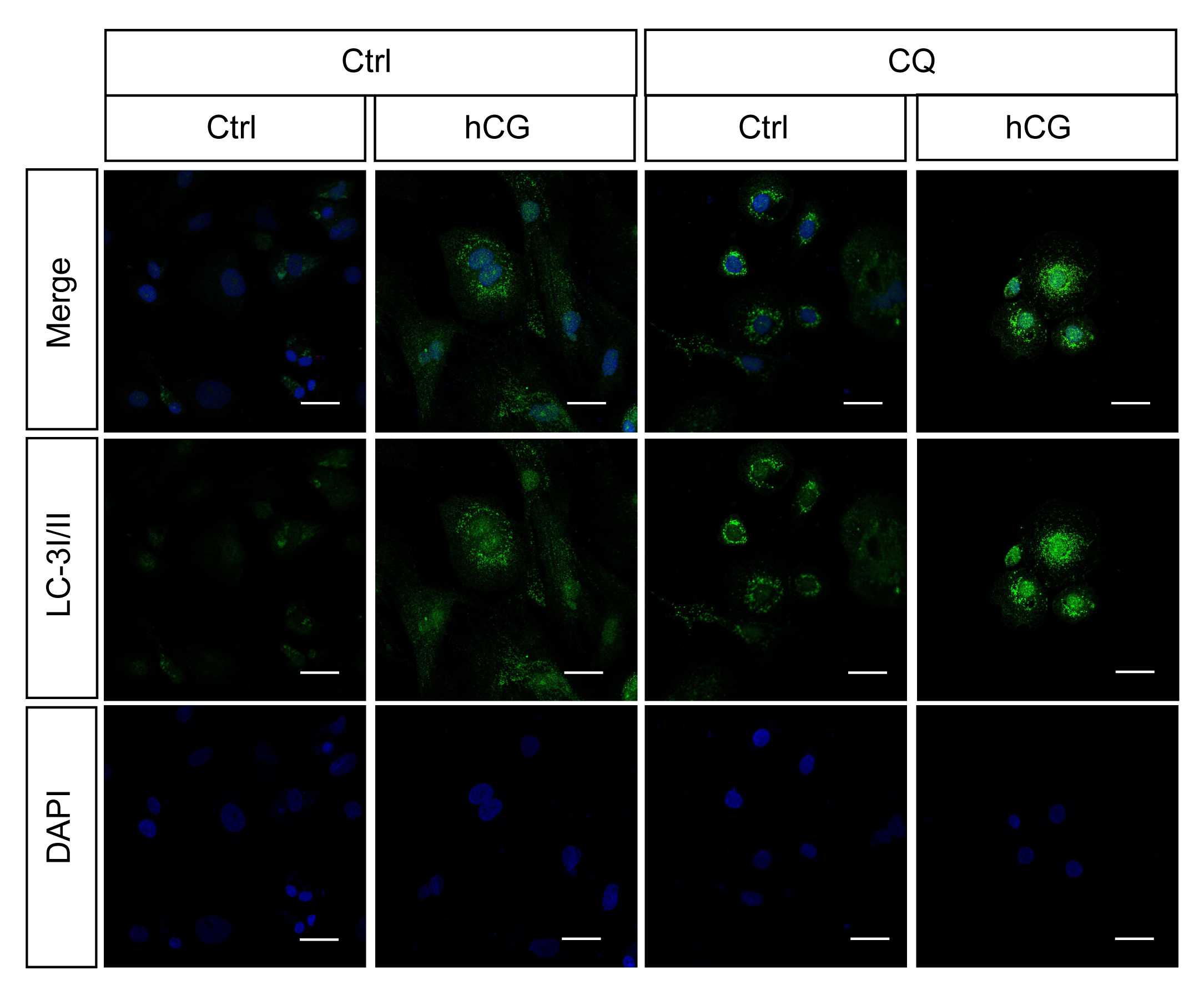


**Figure S1. Effects of hCG and CQ on autophagy induction in granulosa cells.** The granulosa cells were treated with hCG for 48h and then cells were stained with LC-3I/II antibody. To further evaluate autophagy flux, we treated cells with chloroquine (CQ, 50μM) at 24 h before detection. Bar=50μm.

**1.2 Supplementary Figure S2**

**
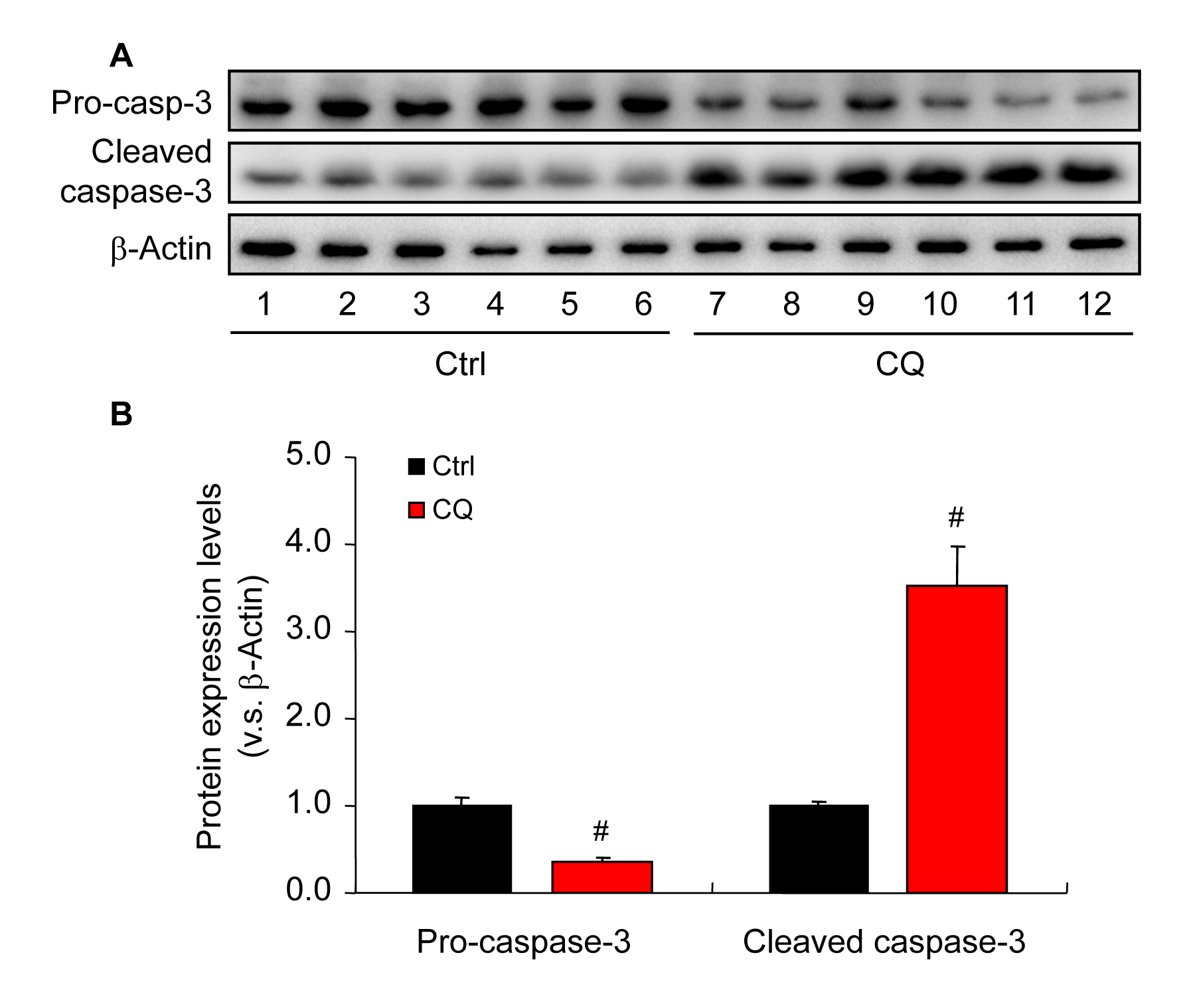
**

**Figure S2 Effect of the inhibition of autophagy on apoptosis during the formation of corpus luteum.** To evaluate the effect of the inhibition of autophagy on apoptosis during the formation of corpus luteum, we treated the rats with chloroquine (CQ, 30 mg/kg body weight) before mating. The expressions of caspase-3 were detected by western blotting. A: Expression changes of pro-caspase-3 and cleaved caspase-3. B: Summarized intensities of pro-caspase-3 and cleaved caspase-3 bloting normalized to the control. Each value represents the mean±SE. One-way analysis of variance (ANOVA) was used to analyze the data, followed by a Tukey’s multiple range test. n=6. #: *P<0.05*, v.s. the Ctrl.

**1.3 Supplementary Figure S3**

**
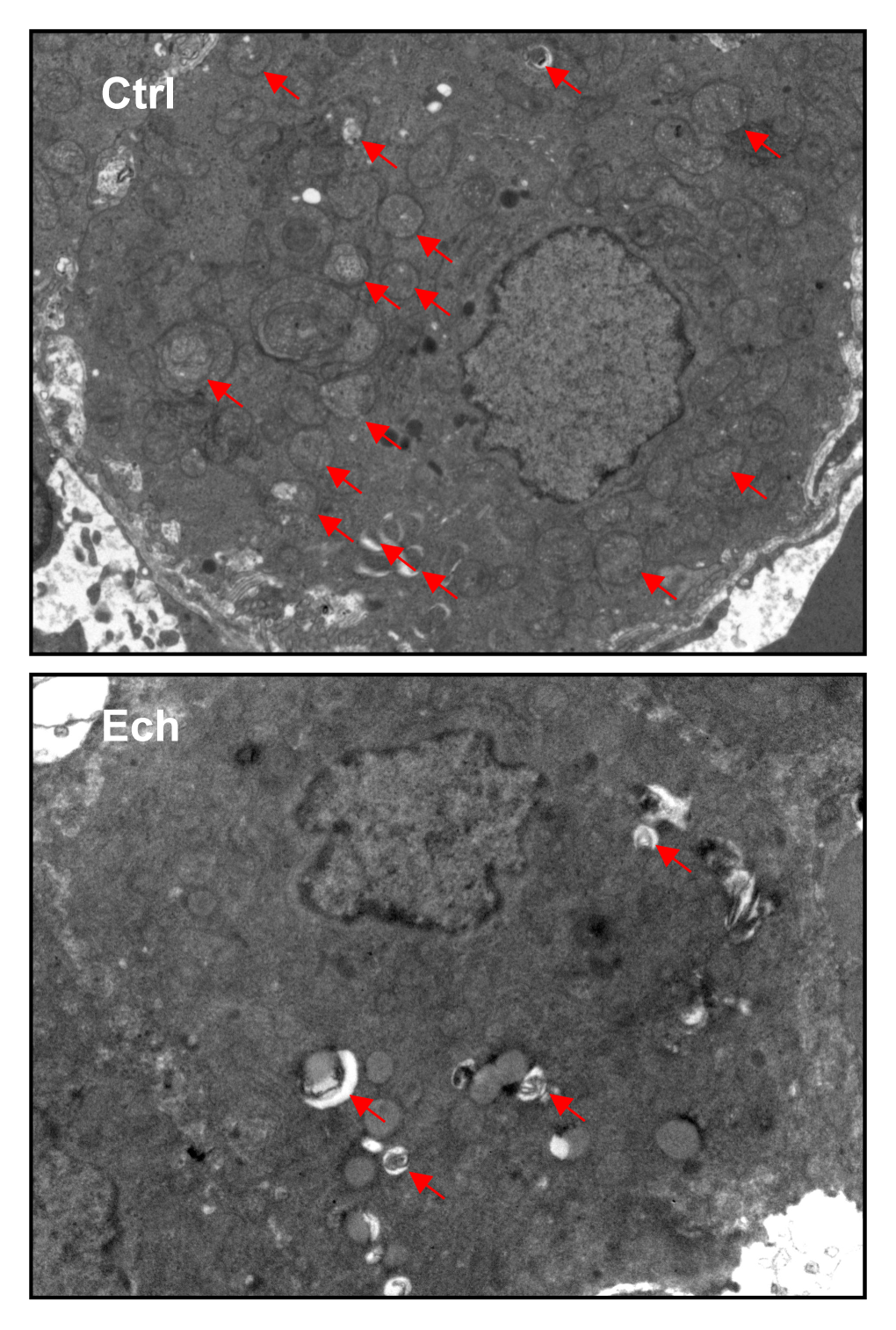
**

**Figure S3 Effect of echinomycin on the formation of autophagosome in whole cell level.** In TEM images, red arrow indicated the autophagosomes in luteal cells with or without echinomycin treatment. Ech: echinomycin

**1.4 Supplementary Table S1**

**Table S**1 Antibody informations for western blotting

| Antibody Name | Company and City | Dilution Degree |
| --- | --- | --- |
| LC-3I/II | Abcam, Cambridge, MA, USA | 1:1000 |
| Beclin1 | Protein Tech Group, Wuhan, China | 1:2000 |
| β-actin | Protein Tech Group, Wuhan, China | 1:5000 |
| LAMP-2 | Protein Tech Group, Wuhan, China | 1:500 |
| p62 | Abcam, Cambridge, MA, USA | 1:1000 |
| cleaved caspase-3 | Cell Signaling Technology, Boston, MA, USA | 1:1000 |
| StAR | Protein Tech Group, Wuhan, China | 1:1000 |
| Bax | Protein Tech Group, Wuhan, China | 1:500 |
| Bcl-2 | Cell Signaling Technology, Boston, MA, USA | 1:1000 |
| COXIV | Protein Tech Group, Wuhan, China | 1:2000 |
| VDAC1 | Protein Tech Group, Wuhan, China | 1:500 |
| anti-cytochrome C | Gene Tex, San Antonio, Texas, USA | 1:2000 |
| HIF-1a | Santa Cruz Biotechnology, Dallas, TX, USA | 1:500 |
| BNIP3 | Abcam, Cambridge, MA, USA | 1:1000 |
| NIX | Abcam, Cambridge, MA, USA | 1:1000 |
| Goat anti-Mouse IgG | Beyotime Institute of Biotechnology, Haimen, China | 1:5000 |
| Goat anti-Rabbit IgG | Beyotime Institute of Biotechnology, Haimen, China | 1:5000 |
